# Synthetic Cyclic C_5_-Curcuminoids Mitigate Oxidative, Apoptotic and Inflammatory Damage in a 6-OHDA-Induced Neuron–Microglia Co-Culture Model of Neuronal Damage

**DOI:** 10.3390/antiox15091085

**Published:** 2026-08-29

**Authors:** Edina Pandur, Zsombor Bence Tóth, Levente Tyukodi, Ilona Gróf, Szilvia Veszelka, Mária A. Deli, Zsuzsanna Rozmer, Imre Huber

**Affiliations:** 1 Department of Pharmaceutical Biology, Faculty of Pharmacy, University of Pécs, Rókus Str. 4, H-7624 Pécs, Hungary; bencetoth1025@gmail.com; 2 Institute of Pharmaceutical Chemistry, Faculty of Pharmacy, University of Pécs, Rókus Str. 4, H-7624 Pécs, Hungary; tyukodi.levente@pte.hu (L.T.); rozmer.zsuzsanna@pte.hu (Z.R.); imre.huber@aok.pte.hu (I.H.); 3 HUN-REN Biological Research Centre, Institute of Biophysics, Temesvári Krt. 62, H-6726 Szeged, Hungary; grof.ilona@brc.hu (I.G.); veszelka.szilvia@brc.hu (S.V.); deli.maria@brc.hu (M.A.D.)

**Keywords:** Parkinson’s disease, blood–brain barrier, curcuminoids, iron, ferroptosis, PARP, NFκB, fractalkine, glutamate

## Abstract

Curcumin and natural curcuminoids possess numerous beneficial properties, including antioxidant, antitumor, and anti-inflammatory effects. However, their application is challenging because of instability and rapid metabolism. Cyclic C_5_-curcuminoids, a subgroup of curcuminoids, are chemically more stable and less susceptible to hydrolysis or metabolism. In our study, seven synthetic compounds—five cyclic C_5_-curcuminoids (compounds **8**, **9**, **11**, **12**, and **13**) and two cyclic chalcones (compounds **4** and **5**)—were examined using an in vitro 6-hydroxydopamine-induced neuronal damage model established with all-trans retinoic acid-differentiated SH-SY5Y and BV-2 microglial cells. The antioxidant, anti-apoptotic (including ferroptosis markers (iron, GSH/GSSG, and malondialdehyde)), apoptotic (PARP, cytochrome c, caspase 9, active caspase-3, and oligonucleosome release), and anti-inflammatory effects of these compounds were also investigated. Compounds **5, 9**, **12**, and **13** significantly decreased reactive oxygen species production (percentage changes: **5**: 28.9%; **9**: 32%; **12**: 29.8%; **13**: 49.2%) and increased antioxidant capacity (percentage changes: **5**: 63.4%; **9**: 85.8%; **12**: 31.2%; **13**: 85.9%) and antioxidant enzyme activity (catalase, superoxide dismutase, and glutathione peroxidase) in SH-SY5Y cells. Compounds **5**, **9**, and **12** decreased microglial activation by downregulating Iba1 expression (fold changes: **5**: 0.73; **9**: 0.39; **12**: 0.33) and decreasing glutamate concentration (percentage changes: **5**: 26.5%; **9**: 41.2%; **12**: 27.3%). Together with compound **13**, they reduced pro-inflammatory cytokine (IL-6, TNFα) secretion and induced anti-inflammatory cytokine (IL-10) release in SH-SY5Y and BV-2 cells. Based on these results, the blood–brain barrier penetrations of compounds **5**, **9**, **12**, and **13** were also investigated. The highest blood–brain barrier penetration was observed for compound **12** (P_app_ value 13.6 × 10^−6^ cm/s). Synthetic cyclic C_5_-curcuminoids derived from curcumin overcome the limitations of stability and bioavailability and exhibit strong antioxidant, anti-inflammatory, and anti-apoptotic effects, making them promising candidates for treating neurodegenerative diseases.

## 1. Introduction

**Figure 1:**
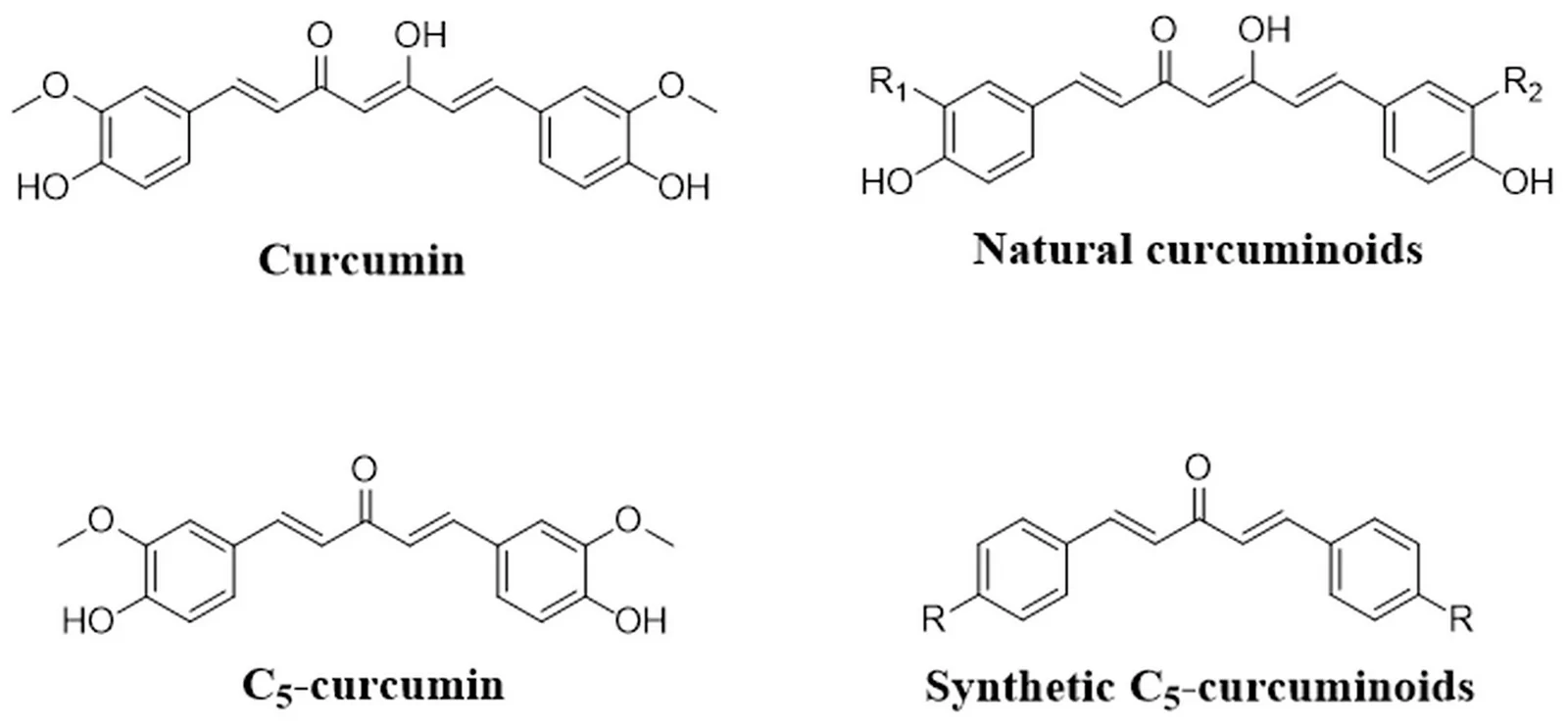
The enol tautomer of the diferuloylmethane dione structure of curcumin. The main natural curcuminoids in turmeric are demethoxycurcumin (R_1_ = OCH_3_; R_2_ = H) and bisdemethoxycurcumin (R_1_ = R_2_ = H). C_5_-curcumin (truncated curcumin) with five carbon atoms between the two aromatic cores is a natural product of *Curcuma longa* L. Synthetic C_5_-curcuminoids with different substituents (R = CH_3_; OCH_3_; N(CH_3_)_2_; Cl or NO_2_) on the aromatic parts. Structural formulas were prepared using the ChemDraw Professional desktop application (version 15.0).

Plants with a long history in traditional medicine are an unbeatable source of lead molecules, offering valuable insights into synthetic strategies in pharmaceutical research and development [1,2]. Curcumin, the main component of turmeric (*Curcuma longa* L. and *Curcuma domestica* L., *Zingiberaceae*), is an excellent example of a compound with diverse pharmacological properties. The isolation of another natural product, C_5_-curcumin, from the same plant was reported in 1993 (Figure 1) [3]. While it remains unclear if C_5_-curcumin is a metabolite of curcumin, the truncated molecule, C_5_-curcumin, can be produced from curcumin through the process of pyrolysis [4].

According to recent review articles, curcumin, as a polyphenol, has shown anti-inflammatory, antiradical, antioxidant, anti-cancer, and multi-drug-resistance-reverting (MDR) activities [5,6,7]. A special scientific focus has been placed on the neuroprotective ability of curcumin [8,9] and in terms of Parkinson’s disease [10]. Studies have also been conducted to demonstrate the neuroprotective efficacy of natural curcuminoids (Figure 1) [11,12]. In addition, curcumin has protective effects on the blood–brain barrier (BBB) in animal and culture models of diseases [13]. However, their medical use is limited by the shortcomings of curcumin and other natural curcuminoids. The most important difficulties with their use are the chemical “(in)stability” (quick metabolism) and poor bioavailability of the structure [13,14].

Another direction in the medicinal chemistry of curcumin is to overcome these limitations by designing and developing more stable analogs of synthetic curcuminoids. An example of this is the neuroprotective synthetic C_5_-curcuminoids. Two of the tested synthetic C_5_-curcuminoids (R = OCH_3_ and R = Cl; Figure 1) improved cognition and reduced the progression of dementia in mice, including in vitro, in vivo tests, and molecular docking validation [15].

**Figure 2:**
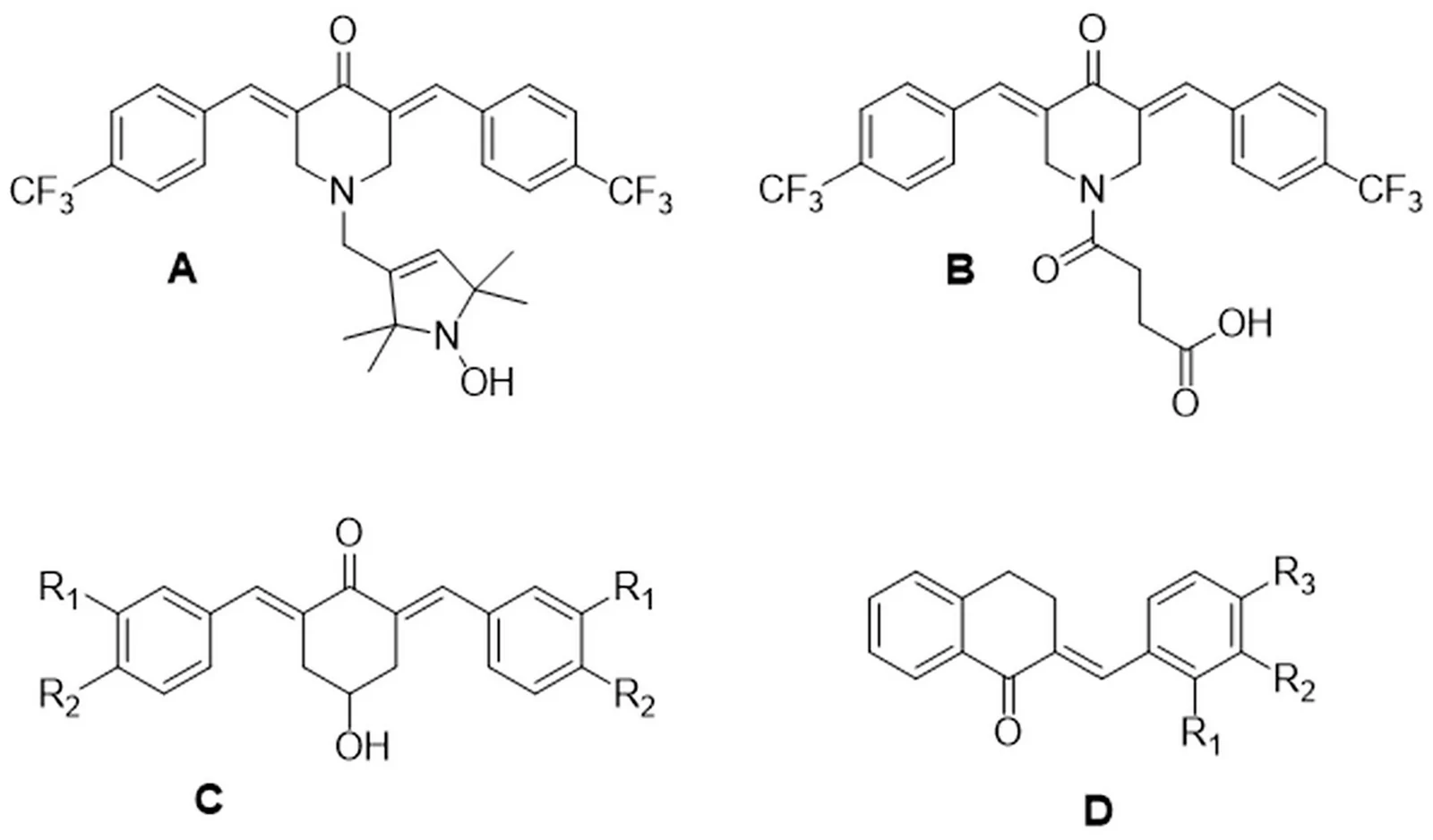
Synthetic homocyclic and heterocyclic C_5_-curcuminoids (**A**–**C**) and synthetic cyclic chalcones (**D**). Structural formulas were prepared using the ChemDraw Professional desktop application (version 15.0).

A further synthetic possibility is the preparation of an even more stable subgroup of curcuminoids, the so-called cyclic C_5_-curcuminoids (Figure 2) [16]. Structure–activity studies (SAR) were performed, for example, in order to find an optimal 3D molecular shape for optimal cytotoxic effect through necessary structural modifications [17,18,19]. The central ring of the synthetic cyclic C_5_-curcuminoids reduces the conformational flexibility to a minimum and constrains the molecules into a “near-to-planar” shape compared to curcumin or C_5_-curcumin. In addition to superior efficacy, synthetic cyclic derivatives have been proven to be chemically more stable and less susceptible to hydrolysis or metabolism [20]. No publications have been identified that discuss synthetic cyclic C_5_-curcuminoids in relation to neurodegenerative diseases, such as Alzheimer’s or Parkinson’s disease, particularly in a neuroprotective context. Therefore, we selected examples from various pharmacological fields to illustrate the chemical stability of these synthetic curcumin analogs.

Compounds **A**, **B**, and **C** (Figure 2) performed well in the different cytotoxicity tests. In addition to its effects on ovarian cancer cells, derivative **A** is a concomitant antioxidant [21]. Compound **B** exhibited high toxicity against neuroblastoma (IC50 = 0.14 nM) and astrocytoma cells (IC50 = 0.39 nM) in parallel and could penetrate the blood–brain barrier [22]. Compounds **C**, the newest subgroup of cyclic C_5_-curcuminoids, hold a unique central ring based on 4-hydroxycyclohexanone and are active antiproliferatives that do not interact with DNA [23]. Synthetic cyclic chalcones (**D**, Figure 2) are a subgroup of the chalcone family and are strongly related to cyclic C_5_-curcuminoids due to their aromatic enone moiety [24]. Moreover, it has also been demonstrated that synthetic C_5_-curcuminoids possess anti-cancer activity in carcinoma cell lines by enhancing oxidative stress, ferroptosis, and apoptosis [25].

As synthetic cyclic C_5_-curcuminoids have not yet been studied in neurodegenerative models, we established a 6-OHDA-induced neuronal damage model using differentiated SH-SY5Y cells. In our previous study, we described the antioxidant effects of novel synthetic cyclic C_5_-curcuminoids. However, their anti-apoptotic and anti-inflammatory effects remained uncharacterized [26]. In the continuation of the study, microglia, the brain’s immune cells, were involved, as these cells play a key role in the process of neurodegeneration by regulating inflammation [27,28,29]. Oxidative stress in the brain affects microglia activation and enhances the release of pro-inflammatory cytokines as well as glutamate from immune cells, leading to neurotoxicity [30,31]. Moreover, activated microglia further increase oxidative stress, leading to severe neuronal damage by increasing the rate of programmed cell death [32,33].

In the present study, we established a co-culture system using all-trans-retinoic acid-differentiated SH-SY5Y cells and BV-2 microglia. The presence of microglia mimics the brain’s immune system, and their effects on neurons can be monitored. Therefore, we demonstrated the beneficial effects of synthetic cyclic C_5_-curcuminoids and chalcones, even in the presence of microglia, which can contribute to neuronal loss. Differentiated SH-SY5Y cells were treated with 6-OHDA to induce an oxidative stress environment, and BV-2 microglia were added to the cultures on sterile coverslips. This model, called bilaminar co-culture, ensures physical interactions between cells and the free diffusion of chemicals.

This study aimed to examine the antioxidant effects of seven synthetic cyclic compounds, cyclic C_5_-curcuminoids (compounds **8**, **9**, **11**, **12**, and **13**) and chalcones (compounds **4** and **5**), in the presence of microglia, considering their modulatory properties. Additionally, we investigated whether these compounds inhibit neuronal death and decrease the production of inflammatory cytokines in both neurons and microglia. Furthermore, the study assessed the ability of synthetic cyclic C_5_-curcuminoids to attenuate the pro-inflammatory effects of microglia and mitigate neuronal damage.

When selecting the compounds, the couples (**4** and **5**, **8** and **9**, or **11** and **12**) formed two chemical groups. One is the phenolic group (**5**, **9**, **12**, and **13**); the other one is the non-phenolic group (**4**, **8**, and **11**). The two groups were also separated pharmacologically in the SAR analysis. In addition, the BBB penetration of compounds **5**, **9**, **12**, and **13** was also determined using a well-established and characterized human blood–brain barrier (BBB) co-culture model [34].

Our objective was to identify a drug candidate with the potential to develop therapy for neurodegenerative disorders.

## 2. Materials and Methods

### 2.1. Chemical Experimental Protocol

A Barnstead-Electrothermal 9100 apparatus (Thermo Fisher Scientific Inc., Waltham, MA, USA) was used to ascertain the uncorrected melting points. Silica gel 60 (0.2–0.5 mm, Merck Life Sciences Kft., Budapest, Hungary) was used for column chromatography, and pre-coated silica gel 60 (F-254, Merck Life Sciences Kft., Budapest, Hungary) plates were used for TLC.

The nuclear magnetic resonance (NMR) ^1^H- and ^13^C-spectra were recorded using a Bruker Avance III 500 (500.15/125.77 MHz for ^1^H/^13^C) spectrometer (Bruker Corp., Ettlingen, Germany) according to our previous publication [22]. The chemical shifts were calibrated against the residual solvent signals. The measurements were conducted at a probe temperature of 298 K using dimethyl sulfoxide (DMSO)-d6 solution. All ^1^H and ^13^C NMR spectra were consistent with the expected structures.

Analyses were performed using a Thermo Dionex UltiMate 3000 HPLC (Dionex, Sunnyvale, CA, USA) system coupled to a Thermo Q Exactive Focus quadrupole-Orbitrap hybrid mass spectrometer (Thermo Fisher Scientific, Waltham, MA, USA).

Chromatographic separation was carried out on a Thermo Accucore RP-MS analytical column (150 × 2.1 mm, 2.6 µm; Thermo Scientific). Eluent A was 0.1% formic acid in water, and eluent B was 0.1% formic acid in methanol. The gradient was 0.0 min, 20% B; 5.0 min, 90% B; 8.5 min, 90% B; and 9.0 min, 20% B, followed by 1.0 min re-equilibration in initial conditions (total run time: 10.0 min). The column was maintained at 40 °C, the flow rate was 0.3 mL/min, and the injection volume was 2 µL. Under these conditions, the analytes were eluted at approximately 4.0 min (**12**), 5.2 min (**13**), 5.3 min (**9**), and 6.0 min (**5**).

The mass spectrometer was equipped with a heated electrospray ionization (HESI) source operating in positive ion mode. The spray voltage was 4.4 kV, capillary (ion transfer tube) temperature was 260 °C, sheath gas flow rate was 30 (arbitrary units), auxiliary gas flow rate was 10 (arbitrary units), and auxiliary gas heater temperature was 300 °C. Full-scan spectra were acquired over *m*/*z* 110–1100 at a resolving power of 35,000 (FWHM at *m*/*z* 200), with an automatic gain control (AGC) target of 3 × 10^6^ and the maximum injection time set to auto.

Each analyte was quantified from the extracted ion chromatogram (EIC) of its protonated molecule [M+H]^+^ using a ±5 ppm mass tolerance: **9**, *m*/*z* 383.1489 (C_22_H_22_O_6_); **5**, *m*/*z* 267.1016 (C_17_H_14_O_3_); **12**, *m*/*z* 368.1493 (C_21_H_21_NO_5_); and **13**, *m*/*z* 468.1653 (C_25_H_25_NO_8_). EICs were extracted using 7-point Gaussian smoothing and integrated using the Genesis algorithm with the default software parameters. Data were acquired and processed with Q Exactive Focus 2.11 and Xcalibur 4.2 software (Thermo Fisher Scientific Inc., Waltham, MA, USA) [26]. The compounds used in this study were resynthesized. Their physical and analytical parameters were identical to those of the previously prepared ones.

### 2.2. Cell Culture Conditions for the BBB Model

Blood was collected from the umbilical cord at Béthune Maternity Hospital (Béthune, France), and CD34+ cells were isolated at the Laboratory of Barrière Hémato-Encéphalique (LBHE), managed by Pr. Maxime Culot. LBHE is the supervisory authority of the Université d’Artois (Arras, France), which is the co-owner of a patent for a human in vitro BBB model based on the use of human CD34+ cells from cord blood co-cultivated with brain pericytes (patent number: WO2014/203087). According to French legislation, all research protocols regarding the collection of umbilical cord blood, isolation of cells, and use of cells derived from human cord blood were approved by the French Ministry of Higher Education and Research (ethical approval number: CODECOH 2011-1321). Written informed consent was obtained from the families before entering the study, stating that umbilical cord blood was collected by Béthune Maternity Hospital and CD34+ cells were isolated by Université d’Artois. CD34+ hematopoietic stem cells were differentiated into endothelial cells (hEC). The Université d’Artois is allowed to negotiate and sign contracts/collaborations with other partners necessary for the exploitation of hEC cells. hEC stocks were purchased from Université d’Artois in the framework of a Material Transfer Agreement between Université d’Artois (Prof. Maxime Culot, Arras, France) and HUN-REN Biological Research Centre (Szeged, Hungary). hEC cells were placed in sterile vials after cryopreservation (approx. 10^6^ CD34+-derived endothelial cells/vial) and transported in a dry ice package from Université d’Artois directly to the recipient laboratory, Prof. Mária A. Deli, HUN-REN Biological Research Centre, and her research group, for the establishment and examination of a BBB co-culture model based on cord blood-derived human endothelial cells in co-culture with brain pericytes in this publication. Throughout the transport, the carrier complied with the regulations governing the transport of biological materials in the EU.

Hematopoietic stem cell-derived human endothelial cells (hEC) [35] were cultured in endothelial cell culture medium (ECM; ScienCell Research Laboratories, Carlsbad, CA, USA) supplemented with 5% FBS (ScienCell Research Laboratories, Carlsbad, CA, USA), 1% endothelial cell growth supplement (ECGS; ScienCell Research Laboratories, Carlsbad, CA, USA), and 50 μg/mL gentamicin (Merck Life Sciences Kft., Budapest, Hungary). For the experiments, hEC (passage number 7) were either grown in co-culture or received conditioned medium from bovine brain pericytes (bPCs) to acquire brain-like properties [35]. Brain pericytes were isolated and maintained as described previously [35] and cultured in 0.2% gelatin-coated 100 mm Costar Petri dishes (Corning Inc., New York, NY, USA) in DMEM (Gibco, Thermo Fisher Scientific Inc., Waltham, MA, USA) supplemented with 20% FBS, 1% GlutaMAX (Gibco, Thermo Fisher Scientific Inc., Waltham, MA, USA), and 50 μg/mL gentamicin. Before the experiments, hEC or the BBB co-culture model was treated with a cARLA cocktail for 2 days [35]. All cultures were maintained in a humidified incubator at 37 °C with 5% CO_2_.

### 2.3. Working Solutions

Stock solutions of 10 mM compounds **5**, **9**, **12**, and **13** were prepared in DMSO (Merck Life Sciences Kft., Budapest, Hungary). Working solutions were diluted in the culture medium to 0.1, 1, and 10 µM concentrations for cell viability experiments. For the permeability experiment, working solutions were diluted in Ringer-Hepes (150 mM NaCl, 5.2 mM KCl, 2.2 mM CaCl_2_, 1.2 mM MgSO_4_, 0.2 mM MgCl_2_, 5 mM D-glucose, 6 mM NaHCO_3_, 10 mM HEPES, pH 7.4) containing 1% bovine serum albumin (BSA) (Merck Life Sciences Kft., Budapest, Hungary) at a concentration of 1 µM, based on cell viability data.

### 2.4. Impedance Measurements by Real-Time Cell Microelectronic Sensing

To measure the cellular response to the compounds, we used an RTCA-SP instrument (Agilent Technologies, Santa Clara, CA, USA). This impedance-based label-free technique has been used by our team for the dynamic monitoring of living cells in real-time and non-invasive quantification of adherent cell proliferation and viability [36]. The RTCA-SP system utilizes 96-well plates containing gold microelectronic sensor arrays (E-plate; Agilent Technologies, Santa Clara, CA, USA). The interaction between cells and electrodes generates an impedance response that correlates linearly with cell number, adherence, and cell growth. This method is more sensitive and informative than colorimetric end-point assays for testing damage or functional changes in brain endothelial cells [37]. The E-plates were coated with collagen IV (100 μg/mL) and fibronectin (25 μg/mL) solution. Culture medium (50 μL) was added to each well for background readings, and 50 µL of human brain endothelial cell suspension was dispensed at a density of 4 × 10^3^ cells/well. The cell index at each time point was defined as (Rn–Rb)/15, where Rn is the cell–electrode impedance of the well containing the cells, and Rb is the background impedance of the well containing the medium alone. The cells were grown for 3–5 days until reaching confluency, which was registered as a plateau phase of the cell index, and then treated with the compounds for 24 h. The results are expressed as normalized cell indices. As a reference compound to induce cell death, TX-100 detergent was used at 1% concentration.

### 2.5. Human Co-Culture Model of the BBB for Permeability Studies

Permeability measurements were performed using a human cell-based BBB co-culture model in which hECs and bPCs were used [35]. To assemble the BBB model, bPCs (passage number < 15) were trypsinized and seeded at the bottom of collagen IV-coated (100 μg/mL) cell culture inserts (polycarbonate membrane, 0.4 μm pore size, 1.12 cm^2^, Corning Inc., New York, NY, USA) at a density of 2 × 10^4^ cells/cm^2^. After seeding, bPCs were allowed to attach to the bottom of the insert for 1 h at 37 °C, and then the insert was turned and placed in a 12-well plate containing endothelial culture medium. hECs were then seeded at a density of 6 × 10^4^ cells/cm^2^ to the other, upper side of the insert coated with collagen IV (100 μg/mL; Merck Life Sciences Kft., Budapest, Hungary) and fibronectin (25 μg/mL; Merck Life Sciences Kft., Budapest, Hungary) in hEC medium. On the fourth day of culture, the luminal compartment of the BBB model was treated with cARLA for 48 h, and experiments were performed on the sixth culture day.

### 2.6. Permeability Experiment

To measure the BBB permeability of compounds **5**, **9**, **12**, and **13** in the luminal-to-abluminal direction mimicking blood-to-brain transfer, the culture inserts were transferred to 12-well plates containing 1500 μL of 0.1% BSA and 0.1% DMSO-Ringer-Hepes buffer in the bottom/acceptor compartments. In the upper/donor compartments, the culture medium was replaced with 500 μL of buffer containing different curcuminoid derivatives at a concentration of 1 µM. To test the function of the BBB model, the flux of permeability marker molecules sodium fluorescein (SF, 10 μg/mL, 376 Da) and Evans blue-labeled albumin (EBA, 10 mg/mL BSA +167.5 μg/mL Evans blue dye, 67 kDa) was also determined. The plates were incubated at 37 °C in 5% CO_2_ for 1 h on a horizontal shaker (~120 rpm). The concentrations of the marker molecules were measured using a Fluorolog-3 fluorescence spectrofluorometer (Horiba Jobin Yvon Inc., Edison, NJ, USA) at 584 nm excitation and 680 nm emission wavelengths for EBA and 485 nm excitation and 520 nm emission wavelengths for SF. After incubation, samples from both compartments were collected in low-retention tubes and analyzed. After the permeability assay, the insert membranes were cut out, and the cells were dissolved in 1 mL of 75% ethanol. Compound concentrations were measured using LC-MS analysis. The quantification of curcuminoid derivatives (LOD and LOQ determinations) is provided in the Supplementary Materials [38].

### 2.7. Calculation of Apparent Permeability Coefficients

The apparent permeability coefficients (P_app_) were calculated as described in our previous study [34]. The cleared volume was calculated from the concentration difference of the derivatives in the bottom/acceptor compartment ([C]_B_) after 1 h and top/donor compartments at 0 h ([C]_A_), the volume of the bottom/acceptor compartment (V_B_; 1.5 mL), and the surface area available for permeability (A; 1.12 cm^2^) using the following equation [34]:

$$P_{app}=\frac{{\left[C\right]}_{B}\times V_{B}}{A\times {\left[C\right]}_{A\;}\times \;t}$$

### 2.8. Calculation of Mass Balance

The mass balance/recovery was calculated from the concentrations of compounds **5**, **9**, **12**, and **13** in the top/donor compartment (Cf_A_), the bottom/acceptor compartment (Cf_B_), the initial concentration of the treatment solutions in the top/donor compartment (C0_A_), and the volumes of the top/donor (VA; 0.5 mL) and bottom/acceptor compartments (V_B_; 1.5 mL) using the following equation [22]:

$$\%Mass\;balance=\frac{C_{fA}V_{A}+C_{fB}V_{B}}{C_{0A}V_{A}}\times 100\%$$

The mass balance was corrected using the concentrations of compounds **5**, **9**, **12**, and **13** in the cells (in 1 mL of 75% ethanol) or remaining absorbed to the cell-free, empty membranes.

### 2.9. Neuron–Microglia Co-Cultures and Treatments

SH-SY5Y human neuroblastoma cells (ATCC-CRL-2266) were maintained in Dulbecco’s Modified Eagle Medium/Nutrient Mixture F-12 medium (DMEM/F12; Capricorn Scientific GmbH, Ebsdorfergrund, Germany). BV-2 murine microglial cells (a gift from Prof. Dr. László Tretter, Semmelweis University, Budapest, Hungary) were cultured in Dulbecco’s Modified Eagle’s Medium (DMEM; VWR International Kft., Debrecen, Hungary). Both cell culture media were supplemented with 10% fetal bovine serum (FBS; Capricorn Scientific GmbH, Ebsdorfergrund, Germany) and 1% penicillin/streptomycin (P/S; Capricorn Scientific GmbH, Ebsdorfergrund, Germany). DMEM/F12 medium also contained 1% non-essential amino acid solution (NEAA, Capricorn Scientific GmbH, Ebsdorfergrund, Germany).

**Figure 3:**
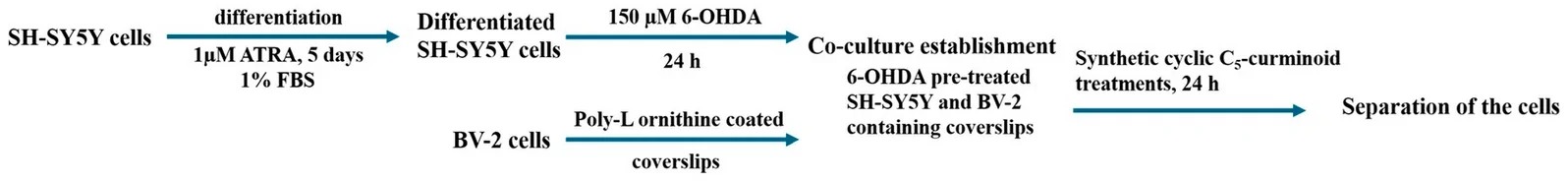
Establishment of the neuron–microglia co-culture.

The SH-SY5Y cells were differentiated using 1 μM all-trans retinoic acid (Merck Life Sciences Kft., Budapest, Hungary) for 5 days in serum-depleted (1% FBS) culture medium. 6-OHDA hydrobromide (Merck Life Sciences Kft., Budapest, Hungary) was dissolved in sterile distilled water to prepare a stock solution (50 mM). Differentiated cells were treated with freshly diluted 150 µM 6-OHDA [26] (Merck Life Sciences Kft., Budapest, Hungary) for 24 h before establishing co-culture systems. BV-2 cells were cultured in poly-L-ornithine (Merck Life Sciences Kft., Budapest, Hungary)-coated culture dishes (Sarstedt AG & Co. KG, Nümbrecht, Germany). During the 24 h pre-treatment of SH-SY5Y cells, BV-2 cells were seeded onto 22 mm sterile coverslips coated with poly-L-ornithine. After an overnight resting period, bilaminar co-cultures were established the following day by turning the BV-2 cells containing coverslips upside down, facing the differentiated and 6-OHDA pre-treated SH-SY5Y cells in fresh 1% serum-depleted DMEM/F12 culture medium (Figure 3). In the bilaminar culture, the cells were separated by a thin layer of culture medium. Therefore, physical and chemical contacts between cells are also possible. The cell cultures were prepared as described in our previous publications [39,40].

The co-cultured cells were treated with seven synthetic cyclic compounds (**4**, **5**, **8**, **9**, **11**, **12**, and **13**) for 24 h. Rasagiline (Merck Life Sciences Kft., Budapest, Hungary), a monoamine oxidase B inhibitor used to treat Parkinson’s disease, was used as the positive control in the experiments at a concentration of 1 µM for 24 h. The optimal treatment concentrations were determined according to the IC50 values (compound **4**: 9.14 ± 1.13 µM; compound **5**: 18.11 ± 2.11 µM; compound **8**: 6.23 ± 1.09 µM; compound **9**: 15.11 ± 1.03 µM; compound **11**: 7.46 ± 1.14 µM; compound **12**: 8.94 ± 1.23 µM; compound **13**: 13.68 ± 1.22 µM) and our previous experiments on differentiated SH-SY5Y cells [26]. The treatment concentrations were selected based on preliminary ROS and TAC studies conducted in our previous publication [26]. The treatment concentrations were as follows: compound **4**, 5 µM; compound **5**, 10 µM; compound **8**, 5 µM; compound **9**, 10 µM; compound **11**, 5 µM; compound **12**, 5 µM; and compound **13**, 10 µM [26]. The control cells were treated with DMSO (Merck Life Sciences Kft., Budapest, Hungary). These concentrations corresponded to approximately 55–80% of the IC50 value for each compound (ranging from 54.7% for compound **4** to 80.3% for compound **8**). They were selected to remain within a pharmacologically active, sub-cytotoxic range for each compound individually, while maintaining consistency with the concentrations used in our previous SH-SY5Y monoculture study. Since the concentrations were neither equimolar across compounds nor matched to an identical IC50 fraction, this design did not support the direct comparison of compound potency.

After the incubation period, the coverslips with BV-2 cells were separated from the surface of the differentiated SH-SY5Y cells, and the two cell types were investigated separately.

### 2.10. Reactive Oxygen Species Measurement

SH-SY5Y cells were cultured in 24-well plates (Biologix Europe, Hallbergmoos, Germany) at a density of 5  ×  10^4^ cells/well. BV-2 cells were cultured on 13 mm poly-L-ornithine-coated coverslips (Thermo Fisher Scientific Inc., Waltham, MA, USA) for 24 h, and one coverslip was added to each well containing the differentiated SH-SY5Y cells as described in Section 2.9.

After treatment and cell separation, ROS levels were measured in SH-SY5Y cells using a Fluorometric Intracellular ROS Kit (Cat. no.: MAK142; Merck Life Technologies Kft, Budapest, Hungary) according to the manufacturer’s instructions. The cells were incubated with 100 µL of ROS reagent for 30 min in a humidified atmosphere at 37 °C. Fluorescence intensity was measured at λex 640 nm/λem 675 nm using an EnSight Multimode microplate reader (PerkinElmer, Rodgau, Germany). ROS levels were compared to those in cells treated with DMSO as a control and expressed as a percentage relative to the control (100%) [25].

### 2.11. Total Antioxidant Capacity Determination

The total antioxidant capacity was measured using the Antioxidant Assay Kit (Cat. no.: MAK334; Merck Life Science Kft, Budapest, Hungary). SH-SY5Y cells were cultured in 6-well plates (Biologix Europe, Hallbergmoos, Germany) at a density of 5  ×  10^5^ cells/well. BV-2 cells were cultured on 22 mm poly-L-ornithine-coated coverslips (Sarstedt AG & Co. KG, Nümbrecht, Germany) for 24 h, and one coverslip was added to each well containing differentiated SH-SY5Y cells. After cell separation, SH-SY5Y cells were collected by centrifugation; then, the pellets were lysed in cold PBS, and 100 µL of each sample was used for measurements. Optical density was measured at 570 nm using a MultiSkan GO spectrophotometer (Thermo Fisher Scientific Inc., Waltham, MA, USA). Antioxidant capacity was expressed as Trolox equivalent in µM [25].

### 2.12. Catalase Assay

Intracellular catalase activity was determined using the Catalase Assay Kit (Cat. no.: MAK531; Merck Life Science Kft., Budapest, Hungary). Differentiated SH-SY5Y cells were cultured in 6 cm dishes (Biologix Europe, Hallbergmoos, Germany) at a density of 10^6^ cells/dish. The co-cultures were established and treated as described above, except that three BV-2 cells containing coverslips were added to one culture dish. After cell separation, SH-SY5Y cells were collected by centrifugation, and the pellets were homogenized on ice in 50 mM potassium phosphate buffer containing 1 mM EDTA. The lysate was centrifuged at 10,000× *g* for 15 min at 4 °C, and the supernatant was used for measurements. After the assay was performed, the optical density was measured at 540 nm using a Multiskan GO spectrophotometer (Thermo Fisher Scientific, Inc., Waltham, MA, USA). Enzyme activity was calculated using the catalase activity equation and expressed in U/L.

### 2.13. Superoxide Dismutase (SOD) Activity Measurement

Differentiated SH-SY5Y cells were cultured in 6 cm dishes (Biologix Europe, Hallbergmoos, Germany) at a density of 10^6^ cells/dish. The co-cultures were established and treated as described above. After cell separation, the SH-SY5Y cells were collected by centrifugation. The Superoxide Dismutase Activity Assay Kit (Cat. no.: MAK528; Merck Life Science Kft., Budapest, Hungary) was used to determine SOD activity according to the manufacturer’s protocol. Briefly, the pellets were homogenized immediately after collection in a cold lysis buffer containing 0.1 M Tris-HCl, 0.5% Triton X-100, 5 mM mercaptoethanol, and protease inhibitors. The supernatants were then used for subsequent reactions. Optical density was measured at 440 nm using a Multiskan GO spectrophotometer (Thermo Fisher Scientific Inc., Waltham, MA, USA). SOD activity was expressed in U/mL [26].

### 2.14. Glutathione Peroxidase (GPx) Assay

Differentiated SH-SY5Y cells were cultured in 6 cm dishes (Biologix Europe, Hallbergmoos, Germany) at a density of 10^6^ cells/dish. The co-cultures were established and treated as described above. Glutathione peroxidase activity was determined using a Glutathione Peroxidase Assay Kit (Cat. no.: MAK437; Merck Life Science Kft, Budapest, Hungary) according to the manufacturer’s protocol. Briefly, SH-SY5Y cells were homogenized in 200 µL of ice-cold 1× PBS by sonication. The supernatants were then used for subsequent reactions. OD was measured at 340 nm using a Multiskan GO spectrophotometer (Thermo Fisher Scientific Inc., Waltham, MA, USA). GPx activity was expressed in U/L [25].

### 2.15. Intracellular Iron Content Determination

Differentiated SH-SY5Y cells were cultured in 6-well plates (Biologix Europe, Hallbergmoos, Germany) at a density of 5  ×  10^5^ cells/well. Co-cultures were established and treated as previously described [41]. After separation, the SH-SY5Y and BV-2 cell pellets were homogenized in 50 mM NaOH at room temperature for 2 h, with gentle shaking. After incubation, the samples were mixed with an equal volume of 10 mM HCl. The samples were then mixed with an iron-releasing reagent (1.4 M HCl, 4.5% *w*/*v*) and incubated for 2 h at 60 °C. For iron detection, an iron detection reagent (6.5 mM ferrozine, 6.5 mM neocuproine, 2.5 M ammonium acetate, and 1 M ascorbic acid) was added to the mixtures and incubated at RT for 30 min. Optical density was measured at 550 nm using a Multiskan GO spectrophotometer (Thermo Fisher Scientific Inc., Waltham, MA, USA). A standard curve of FeCl_3_ was used to determine the iron concentration. The protein concentration was determined for each sample using a DC Protein Assay Kit (Cat. no.: 5000112; Bio-Rad Inc., Hercules, CA, USA) to normalize the iron levels, and the intracellular iron content was expressed as µM iron/mg protein [25].

### 2.16. Glutathione (GSH/GSSG) Measurement

Differentiated SH-SY5Y cells were cultured in 6 cm dishes (Biologix Europe, Hallbergmoos, Germany) at a density of 10^6^ cells/dish. The co-cultures were established and treated as described above. The oxidized glutathione (GSSG) and total glutathione concentrations were measured in differentiated SH-SY5Y cells using the Glutathione GSH/GSSG Assay Kit (Cat. no.: MAK440; Merck Life Science Kft, Budapest, Hungary). After separation of the cells, the SH-SY5Y cell pellets were lysed in cold buffer containing 50 mM phosphate, pH 7, 1 mM EDTA, and 20 µL of the Scavenger for GSSG measurement. To determine the total glutathione concentration, the cells were homogenized in a cold buffer containing 50 mM phosphate (pH 7) and 1 mM EDTA. After centrifugation, the supernatants were deproteinated using a 5% meta-phosphoric acid solution. Following centrifugation at 14.000× *g* for 5 min, 6 µL of the supernatant was used to detect glutathione. Optical density was measured at 412 nm wavelength. The total, oxidized, and reduced glutathione concentrations were calculated using the equations provided in the protocol.

### 2.17. Malondialdehyde Measurements

MDA levels were measured using the Malondialdehyde (MDA) Colorimetric Assay Kit (Cat. no.: EEA015; Thermo Fisher Scientific Inc., Waltham, MA, USA). Differentiated SH-SY5Y cells were cultured in 6 cm dishes (Biologix Europe, Hallbergmoos, Germany) at a density of 10^6^ cells/dish. Co-cultures were established and treated as described above. After separation, SH-SY5Y cells were collected and lysed in PBS. After centrifugation, the supernatants were used for MDA detection. Optical density was determined at 532 nm using a Multiskan GO spectrophotometer. The MDA concentration was calculated using a standard curve (0–40 μmol). The results were normalized to the protein content of the supernatant and expressed as μmol/g protein [26].

### 2.18. Glutamate Measurement

Differentiated SH-SY5Y cells were cultured in 6-well plates (Biologix Europe, Hallbergmoos, Germany) at a density of 5  ×  10^5^ cells/well. Co-cultures were established and treated as previously described. Intracellular and extracellular glutamate levels were determined using the Glutamate Assay Kit-WST (Cat. no.: G269; Dojindo EU GmbH, Munich, Germany). After separation, the SH-SY5Y and BV-2 cell pellets were homogenized in Lysis Buffer. After centrifugation, the supernatants were concentrated, and 40 µL of the samples was used for measurements. After adding the working solution, the samples were incubated at 37 °C for 30 min. Extracellular glutamate levels were determined using the supernatant of the co-cultures the same way as in the case of cell lysates. Absorbance was measured at 450 nm. The glutamate levels of the samples were calculated using a glutamate standard curve, and the concentrations were expressed in mM.

### 2.19. Real-Time PCR

Co-cultures were established and treated as previously described. After separation, BV-2 cells were collected from the coverslips using trypsinization and centrifugation. Total RNA was isolated from BV-2 cells using the Aurum Total RNA Isolation Kit (Cat. no.: 7326820; Bio-Rad Inc., Hercules, CA, USA), and complementary DNA (cDNA) was synthesized using the iScript cDNA Kit (Cat. no.: 1708891; Bio-Rad Inc., Hercules, CA, USA) according to the manufacturer’s protocol. Real-time PCR was performed using the Opus 96 Real-Time System (Bio-Rad Inc., Hercules, CA, USA) with iTaq Universal SYBR Green Supermix (Cat. no.: 1725121; Bio-Rad Inc., Hercules, CA, USA). The specificity of each reaction was validated by generating melting curves (Supplementary Figure S1). Relative gene expression was calculated using the Bio-Rad CFX Manager 3.1 software (Bio-Rad Inc., Hercules, CA, USA). For normalization, the β-actin housekeeping gene was used. Relative expression rates were expressed as fold changes relative to those of the control. The gene expression of the controls was regarded as 1 [25]. The primer sequences used in this study are presented in Table 1.

### 2.20. Enzyme-Linked Immunosorbent Assay (ELISA)

The co-cultures were treated as described above. After the treatments, the cell culture medium, SH-SY5Y, and BV-2 cells were collected and stored at −80 °C until analysis. Intracellular cleaved (inactive) poly ADP-ribose polymerase, mono- and oligonucleosomes, cytochrome c, caspase-9, and cleaved caspase-3 concentrations were measured in differentiated SH-SY5Y cells using the Human PARP (Cleaved) [214/215] ELISA Kit (Cat. no.: KHO0741; Thermo Fisher Scientific Inc., Waltham, MA, USA), the Cell Death Detection ELISA^PLUS^ (Cat. no.: CELLDETH-RO; Merck Life Science Kft, Budapest, Hungary), the Human Cytochrome C ELISA Kit (Cat. no.: BMS263; Thermo Fisher Scientific Inc., Waltham, MA, USA), the Human Caspase 9 ELISA Kit (Cat.no.: BMS2025; Thermo Fisher Scientific Inc., Waltham, MA, USA) and the Human Caspase 3 (Cleaved) ELISA Kit (Cat. no.: KHO1091; Thermo Fisher Scientific Inc., Waltham, MA, USA) according to the manufacturer’s protocol. Cytokine measurements were performed using the supernatants. Based on the specificity tests provided by the manufacturer, the ELISA kits used are specific for the human or mouse cytokines being tested. The human (SH-SY5Y)- and mouse (BV-2)-originated cytokines were determined using the Human IL-10 ELISA Kit (Cat. no.: BMS215-2), the Mouse IL-10 ELISA Kit (Cat. no.: BMS614), the Human IL-6 ELISA Kit (Cat. no.: KHC0061), the Mouse IL-6 ELISA Kit (Cat. no.: KMC0061), the Human TNFα ELISA Kit (Cat. no.: BMS223-4), and the Mouse TNFα ELISA Kit (Cat. no.: BMS607-3). All cytokine ELISA kits were purchased from Thermo Fisher Scientific, Inc. (Waltham, MA, USA). Fractalkine concentrations were measured in the cell culture supernatants using the Human Fractalkine ELISA Kit (Cat. no.: EHCX3CL1; Thermo Fisher Scientific Inc., Waltham, MA, USA). Optical density was measured using a Multiskan GO spectrophotometer (Thermo Fisher Scientific Inc., Waltham, MA, USA) at a wavelength of 450 nm. The concentrations of the target cytokines were calculated using the SkanIt RE 5.0 software. Cyt c and Cleaved PARP levels were expressed as ng/mL, and caspase-9, cleaved caspase-3, and cytokine concentrations were expressed as pg/mL.

### 2.21. Western Blot

For Western blot analysis, differentiated SH-SY5Y cells were cultured in 6 cm dishes (Biologix Europe, Hallbergmoos, Germany) at a density of 10^6^ cells/dish. The co-cultures were established and treated as described above. After cell separation, SH-SY5Y and BV-2 cell pellets were lysed in cold lysis buffer (50 mM Tris-HCl, 150 mM NaCl, 0.5% Triton X-100, pH 7.4). After centrifugation, the protein concentrations were measured using a DC Protein Assay Kit (Cat. no.: 5000112; Bio-Rad Inc., Hercules, CA, USA). Equal amounts of protein samples were separated by SDS-PAGE using a Mini-Protean Tetra Cell system (Bio-Rad Inc., Hercules, CA, USA). The proteins were then transferred to nitrocellulose membranes using a Trans-Blot Turbo Transfer System (Bio-Rad Inc., Hercules, CA, USA). The membranes were blocked with EveryBlot Blocking Buffer (Bio-Rad Inc., Hercules, CA, USA) for 5 min. The following primary antibodies were used in the experiment: anti-NFκB1 p105/p50 IgG (Cat. no.: 12540; 1:1000; overnight at 4 °C, gentle shaking; Cell Signaling Technology Europe, Leiden, The Netherlands) and anti-phospho-p65 IgG (Cat. no.: 3033; 1:1000; overnight at 4 °C, gentle shaking; Cell Signaling Technology Europe, Leiden, The Netherlands). Anti-GAPDH IgG (Cat. no.: G9545; 1:3000; 1 h RT, gentle shaking; Merck Life Science Kft, Budapest, Hungary) was used as the loading control for SH-SY5Y cells, and β-actin (Cat. no.: A5316; 1:2000; 1 h RT, gentle shaking; Merck Life Science Kft, Budapest, Hungary) was used as the loading control for BV-2 cells. Goat anti-rabbit IgG antibody (1:12,000; 1 h, RT, gentle shaking; Merck Life Science Kft., Budapest, Hungary) was used as the secondary antibody. WesternBright ECL chemiluminescent substrate was used to detect the protein bands. The UVITec Alliance Q9 System (UVITec, Cambridge, UK) was used for imaging. NineAlliance Q9 UVITec software (v18.16c ) was used for data analysis. The expression of the target protein was expressed as the percentage of the target to housekeeping protein ratio [25].

### 2.22. Data Analysis

BBB/permeability data: In the cell impedance and BBB penetration studies, all data are presented as mean  ±  standard deviation (SD). The number of independent experiments was 4–6 (BBB) and 6–8 (cell impedance). The values were compared using one-way analysis of variance (ANOVA) followed by Bonferroni or Dunnett’s test using GraphPad Prism 5.0 software (GraphPad Software Inc., Boston, MA, USA). Changes were considered statistically significant at *p* < 0.05.

Cellular assays: Three biological replicates were used for each cellular assay. Five technical replicates were used for ROS measurements, and three technical replicates were used for TAC, Cat, GPx, SOD, intracellular iron, GSH/GSSG ratio, MDA, real-time PCR, and ELISA measurements. Western blot images are representative of the three independent experiments. Statistical analyses were performed using the SPSS software (version 24.0; IBM Corporation, Armonk, NY, USA). Statistical significance was determined using one-way ANOVA and Tukey’s post hoc tests. Data are presented as the mean  ±  SD. The level of significance was set at *p* < 0.05. Significant differences compared to the controls are indicated by an asterisk (*), and significant alterations compared to the 6-OHDA-treated cells are indicated by a cross symbol (†).

## 3. Results

### 3.1. Chemical Synthesis

**Figure 4:**
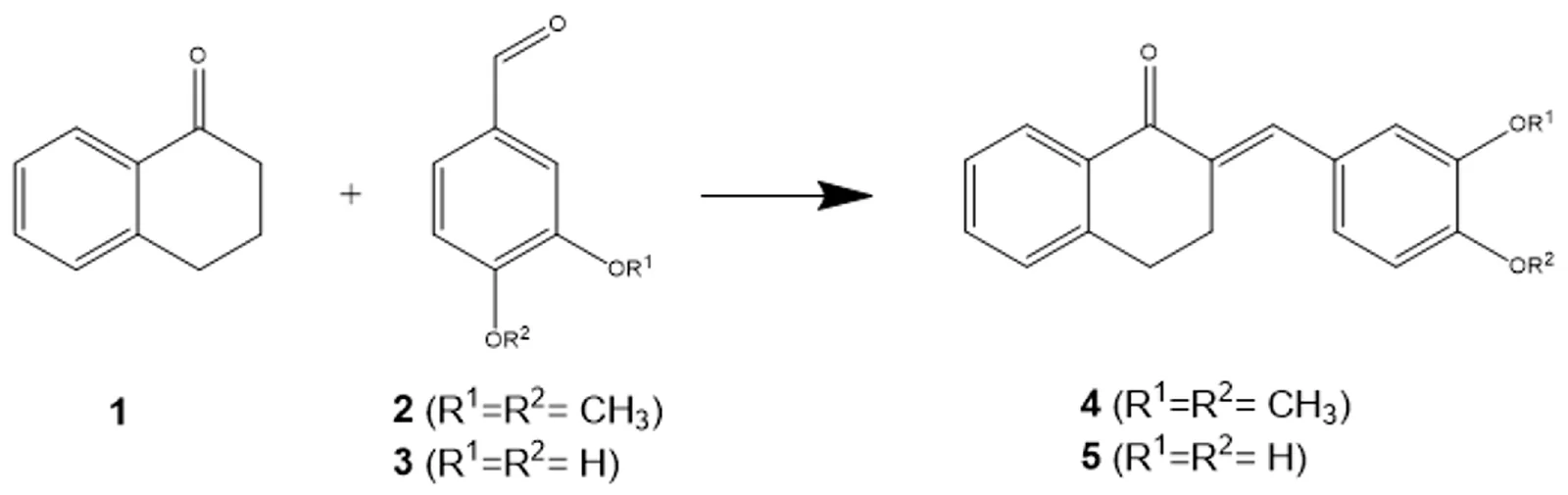
Synthetic pathway for the synthesis of cyclic chalcone analogs **4** and **5** [26]. A Claisen–Schmidt condensation was performed starting from tetralone (**1**) in the presence of aromatic aldehyde **2** for the dimethoxy compound **4** in basic medium, and with aldehyde **3** for the diphenol derivative **5** in acidic medium. Structural formulas were prepared with the ChemDraw Professional desktop application.

**Figure 5:**
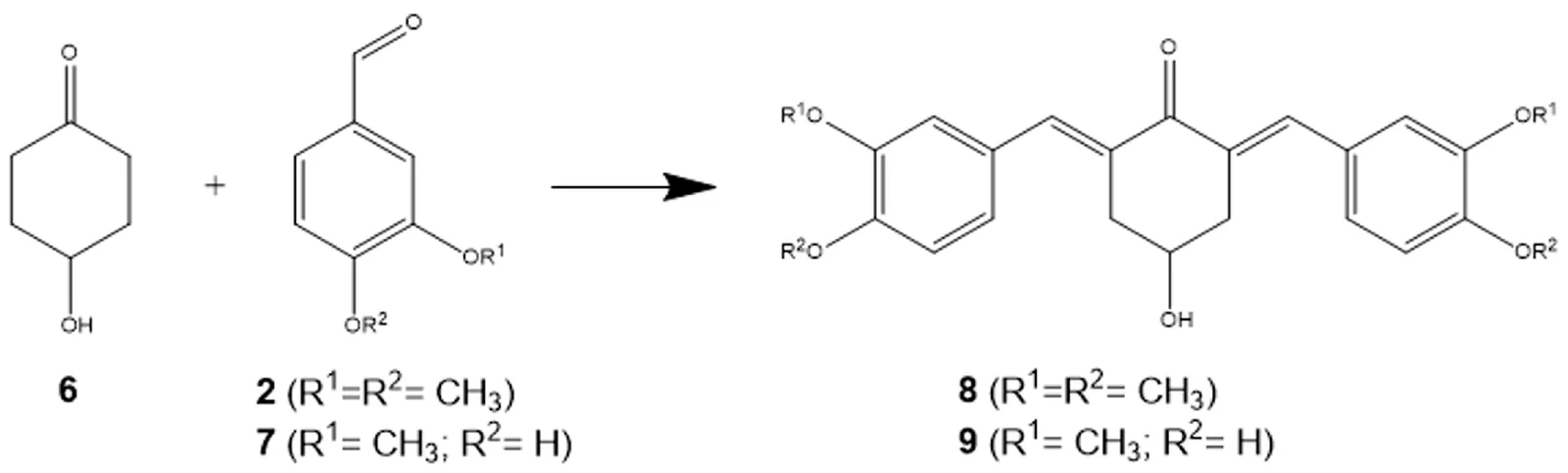
Synthesis of cyclic C_5_-curcuminoids **8** and **9**: 4-hydroxy-cyclohexanone (**6**) was used in the base-catalyzed Claisen–Schmidt condensation for **8** and in the acid-catalyzed one for **9** [26]. Structural formulas were prepared with the ChemDraw Professional desktop application.

**Figure 6:**
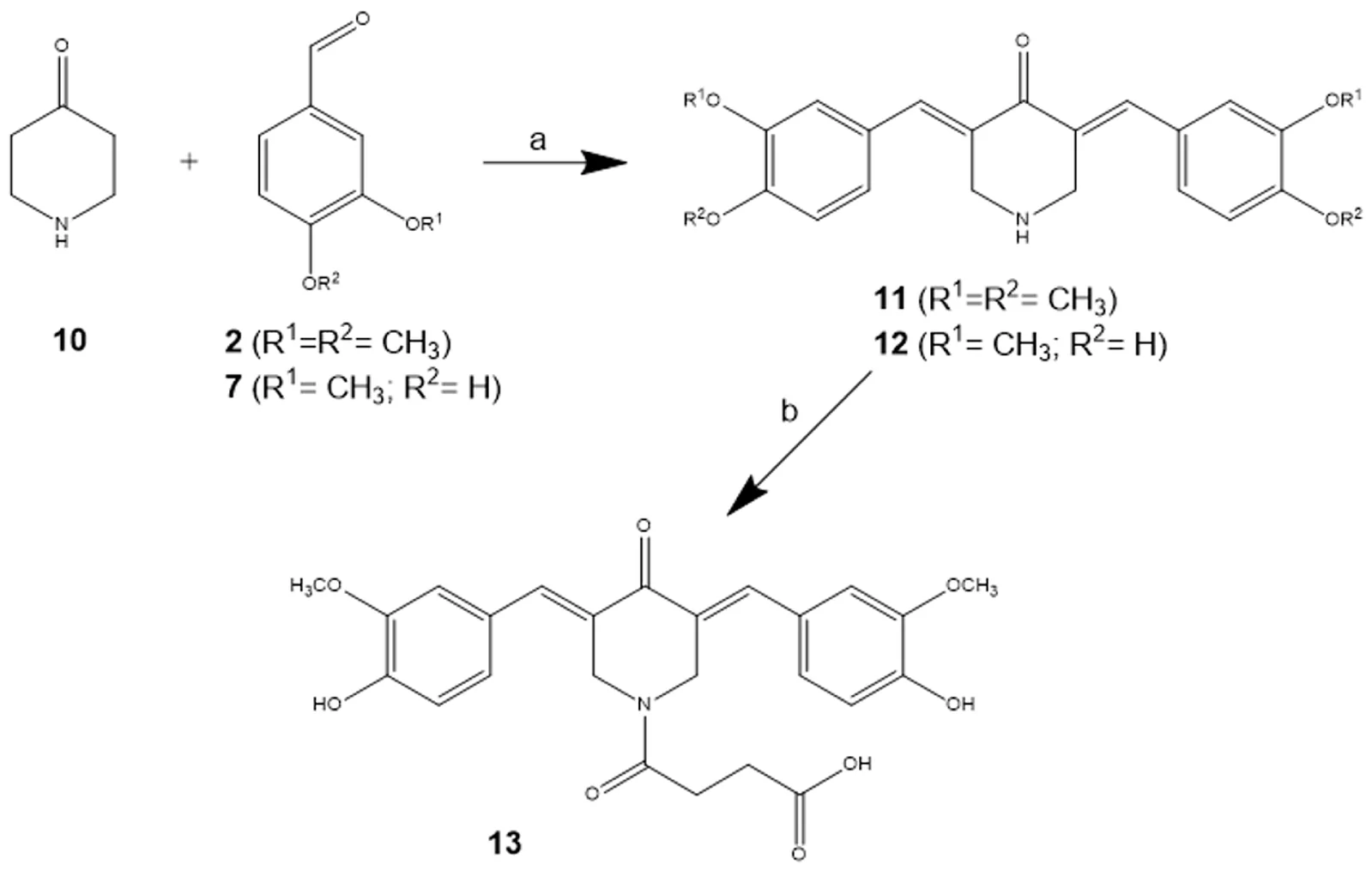
Both cyclic C_5_-curcumin derivatives **11** and **12** were obtained via acid-catalyzed Claisen–Schmidt condensation (a) [26]. Compound **12** was transformed into **13** via an acylation reaction with succinic anhydride in the presence of triethylamine (b). Structural formulas were prepared with the ChemDraw Professional desktop application.

The synthesis of targeted cyclic C_5_-curcuminoid (**8**, **9**, **11**, **12**, and **13**) and cyclic chalcone (**4** and **5**) derivatives for this study was performed according to our previous publication [26]. All reagents, reaction conditions, and methods were identical to those previously published. To simultaneously observe the corresponding structures and numbering, we provided the reaction pathways (Figure 4, Figure 5 and Figure 6).

### 3.2. Effect of Compounds 5, 9, 12, and 13 on the Impedance of Brain Endothelial Cells

**Figure 7:**
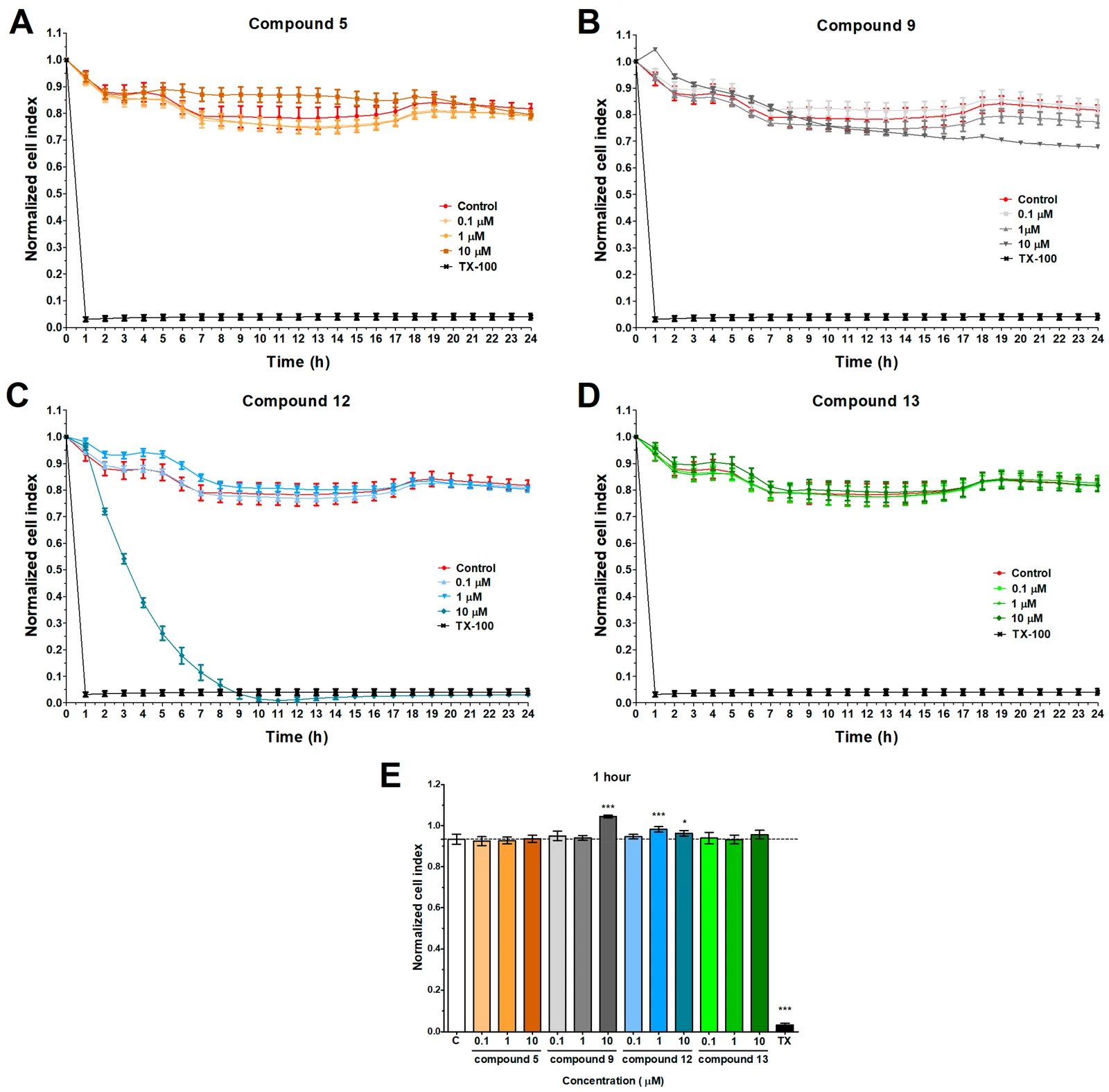
The effect of different concentrations of (**A**) compound **5**, (**B**) compound **9**, (**C**) compound **12**, and (**D**) compound **13** on the impedance kinetics of human brain endothelial cell monolayers. (**E**) Effect of the tested curcuminoids at 1 h time point. Values are presented as mean ± SD, *n* = 6–8. Statistical analysis was performed using one-way ANOVA followed by Dunnett’s test. * *p* < 0.05, *** *p* < 0.001 compared to control. Abbreviations: C, control; TX, TX-100.

The four curcuminoid derivatives were tested for the capability to penetrate the BBB. As a first step, the impedance of brain endothelial cell layers was determined (Figure 7), similarly to our previous study [22], which reflects barrier integrity as well as cell response and viability. The impedance profiles remained similar to the control group (shown in red in Figure 7A–D) for most compounds and concentrations, indicating good barrier properties and cell viability. At the 1 h time point (Figure 7E), the 10 µM concentration of compound **9** and the 1 and 10 µM concentrations of compound **12** slightly and transiently increased the cell index values. Since impedance values reflect several cellular parameters simultaneously, including cell adhesion, morphology, membrane properties, and cell–cell contacts, giving a complex biological readout, the small transient impedance elevation can be interpreted either as improved barrier tightness, based on our previous study [34], or membrane-associated changes. However, at later time points, the highest 10 µM concentration of compound **12** decreased cell impedance (Figure 7C) and, after 8 h incubation, caused cell death. Based on these results, permeability experiments were performed at a concentration of 1 µM for all the curcuminoid derivatives.

### 3.3. Permeability of Compounds 5, 9, 12, and 13 Across the Human Cell-Based BBB Model

**Figure 8:**
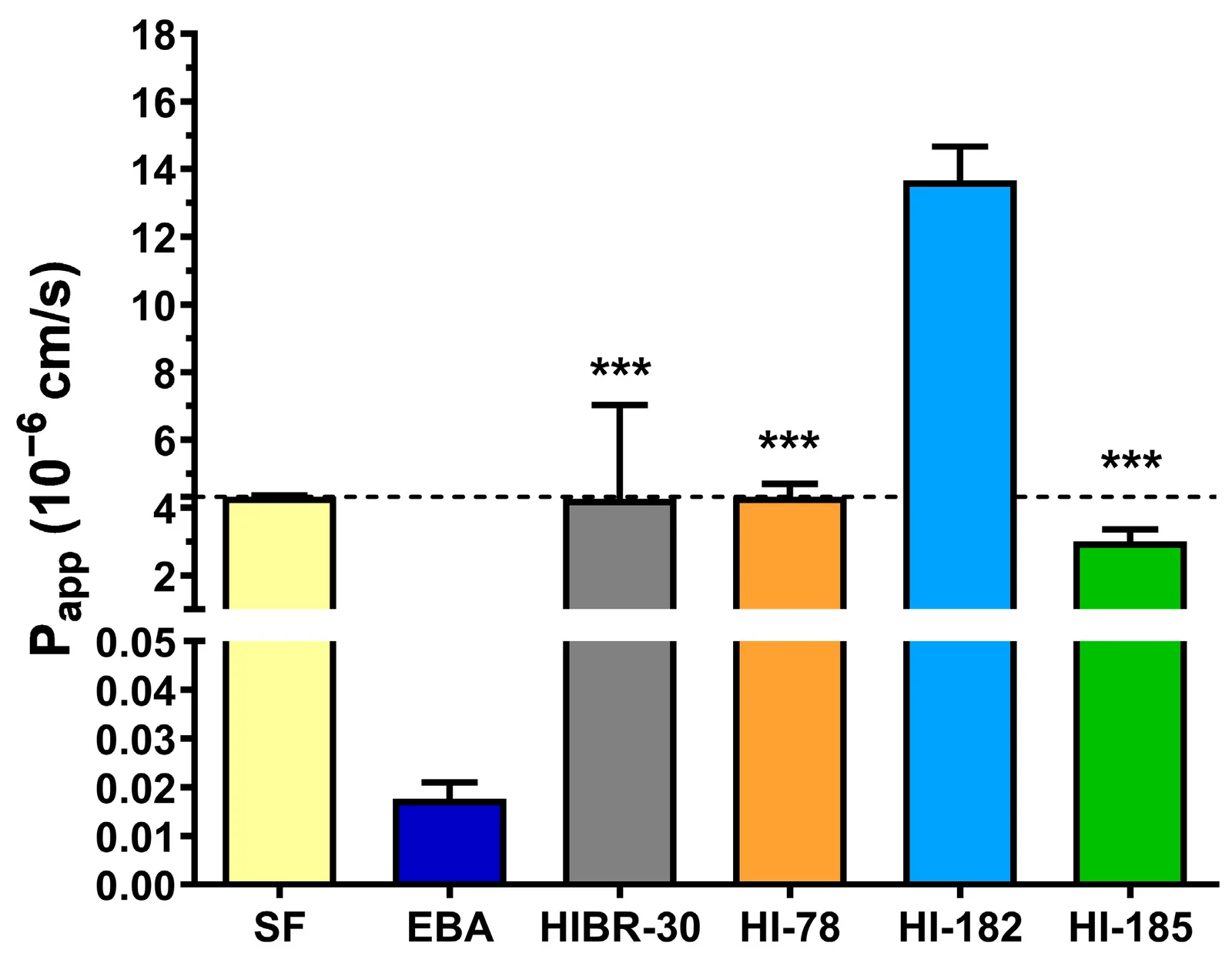
Permeability of compounds **5**, **9**, **12**, and **13** (1 μM) across the culture model of the BBB after 1 h of treatment. Columns represent mean ± SD, *n* = 4–6. Statistical analysis was performed using one-way ANOVA followed by the Bonferroni test. *** *p* < 0.001 compared to SF. The dotted line indicates the average permeability of the SF permeability marker molecule. Abbreviations: P_app_, apparent permeability coefficient; SF, sodium fluorescein (376 Da); EBA, Evans blue–albumin complex (67 kDa).

The human BBB model showed good barrier properties based on the low P_app_ values for passive paracellular BBB permeability marker molecule SF and transcellular permeability marker EBA (Figure 8), similarly to our previous study characterizing the model [34]. Compound **12** exhibited the highest penetration, 13.6 × 10^−6^ cm/s, across the BBB model among the four compounds, which was 3-fold higher than the P_app_ of sodium fluorescein (SF) (Figure 8). Based on our previous results and expertise [42], P_app_ values above 10–15 × 10^−6^ cm/s can be considered as good BBB penetration. The penetration of compounds **9** (4.3 × 10^−6^ cm/s), **5** (4.3 × 10^−6^ cm/s), and **13** (3 × 10^−6^ cm/s) was in the range of the P_app_ of the marker molecule sodium fluorescein (SF; Figure 8). These results indicate the low passive diffusion of curcuminoid derivatives **5**, **9**, and **13** across the human BBB model.

The penetration of the four compounds was determined in cell-free inserts, too. High P_app_ values were measured for all the derivatives (compound **5**: 34.6 × 10^−6^ cm/s; compound **9**: 22.6 × 10^−6^ cm/s; compound **12**: 45.8 × 10^−6^ cm/s; compound **13**: 28.3 × 10^−6^ cm/s), indicating that the membranes of the inserts were highly permeable to the molecules and indeed the human BBB model was responsible for the low penetration.

After the BBB permeability experiments, recovery/mass balance values were also determined (Table 2). The recovery/mass balance value of 70% of compound **13** can be considered good in drug permeability assays [42]. For compounds **5**, **9**, and **12**, low recovery values were measured (Table 2). No cellular uptake was detected for compounds **9** and **13**, and less than 3% of compounds **5** and **13** were measured in the cell fraction at the end of the permeability experiment, indicating that the tested derivatives did not enter and were trapped in the brain endothelial cells in significant amounts (Table 2).

As a control experiment, the recovery values of the tested derivatives were calculated using only cell-free inserts. Two curcuminoid derivatives showed high recovery values ≥ 70% (compounds **12** and **5**), indicating that they did not bind to the membrane of the inserts or the plastic surfaces (Table 2). However, these derivatives showed low recovery values measured in the BBB model; therefore, we speculate that these molecules can be metabolized by brain endothelial cells. The recovery value of compound **13** was within the acceptable range for both the BBB model and cell-free inserts. The recovery values were the lowest for compound **9**, measured on both cell-free inserts (63%) and the BBB model (37%), indicating binding to the membrane of the inserts and/or plastic surfaces, as well as metabolism by the cells.

### 3.4. Cyclic C_5_-Curcuminoids and Chalcones Reduce the Production of ROS by Increasing Antioxidant Capacity in Co-Cultured SH-SY5Y Cells

**Figure 9:**
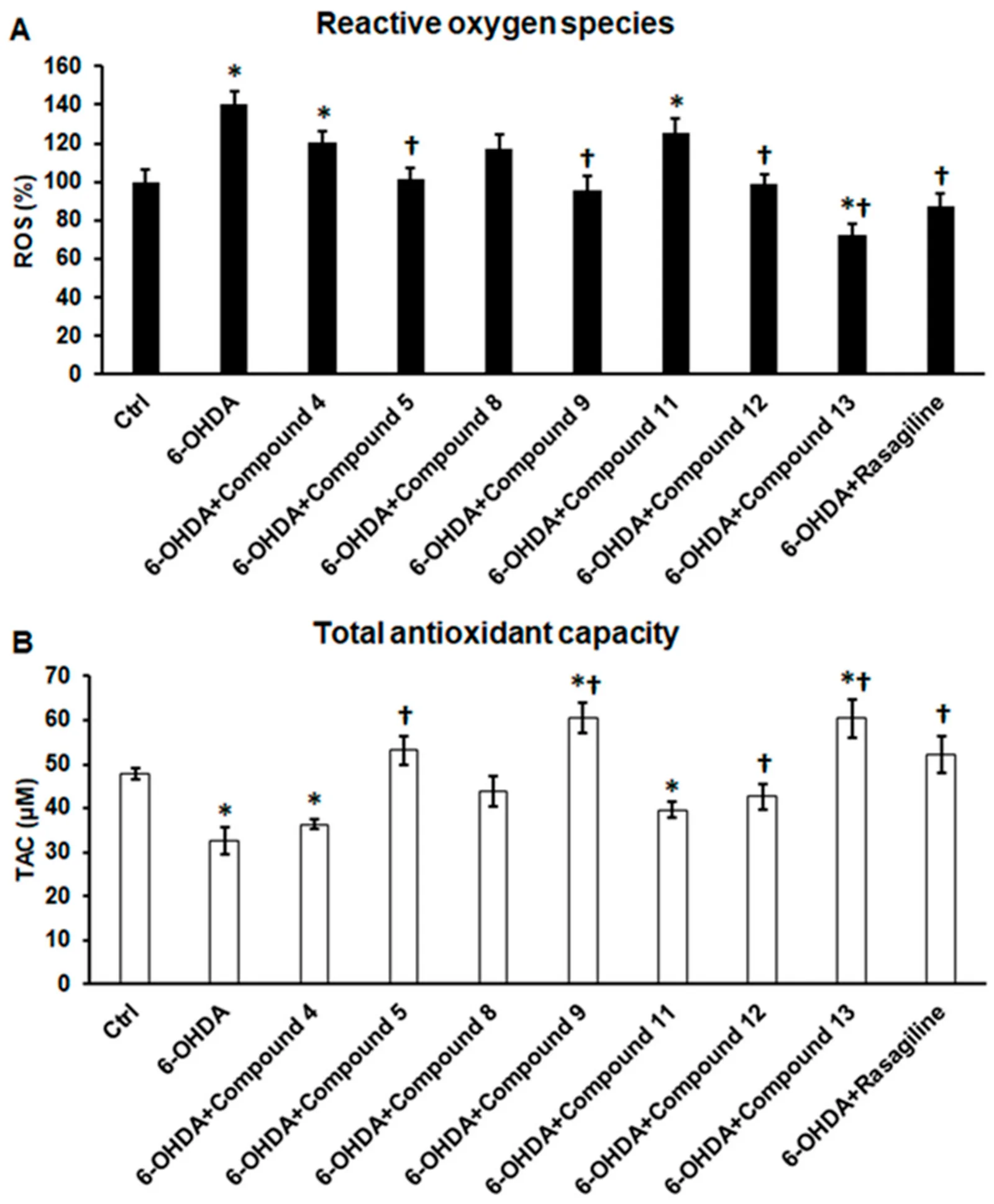
Determination of reactive oxygen species (**A**) and total antioxidant capacity (**B**) in co-cultured differentiated SH-SY5Y cells. Oxidative stress was evaluated by 6-OHDA treatment of SH-SY5Y cells. The cells were then treated with different synthetic C_5_-curcuminoids or rasagiline, and BV-2 microglia were added to the cultures. After cell separation, ROS levels were determined in SH-SY5Y cells using a Fluorometric Intracellular ROS Kit. ROS values were expressed as percentages of the control cells. The total antioxidant capacity of the neurons was determined using the Antioxidant Assay Kit. TAC was expressed as Trolox equivalents (µM). The columns represent the mean values ± standard deviation of three independent experiments. The asterisk shows *p* < 0.05 compared to the control, and the cross indicates *p* < 0.05 compared to the 6-OHDA treatment.

6-OHDA treatment significantly increased ROS production and decreased total antioxidant capacity (TAC), suggesting the establishment of oxidative stress (Figure 9A,B).

Compounds **5** (*p* = 0.002), **9** (*p* = 0.0016), **12** (*p* = 0.0013), and **13** (*p* = 0.0002) significantly reduced ROS production compared to 6-OHDA treatment (Figure 9A). Moreover, compound **13** (*p* = 0.0053) significantly reduced ROS levels compared to those in the control (Figure 9A). In contrast, these compounds significantly increased the total antioxidant capacity of the neurons compared to 6-OHDA treatment, and compounds **9** (*p* = 0.0039) and **13** (*p* = 0.0082) showed higher TAC levels than the control (Figure 9B).

Compounds **4**, **8**, and **11** did not reverse the effects of 6-OHDA, suggesting that these compounds do not provide antioxidant protection to SH-SY5Y cells (Figure 9A,B).

### 3.5. Cyclic C_5_-Curcuminoids and Chalcones Modify the Activity of the Antioxidant Enzymes in the Co-Cultured SH-SY5Y Cells

Considering that TAC evaluates the antioxidant defense provided by both small molecules and enzymes, our objective was to determine the individual contributions of catalase, superoxide dismutase, and glutathione peroxidase antioxidant enzymes to this defense.

**Figure 10:**
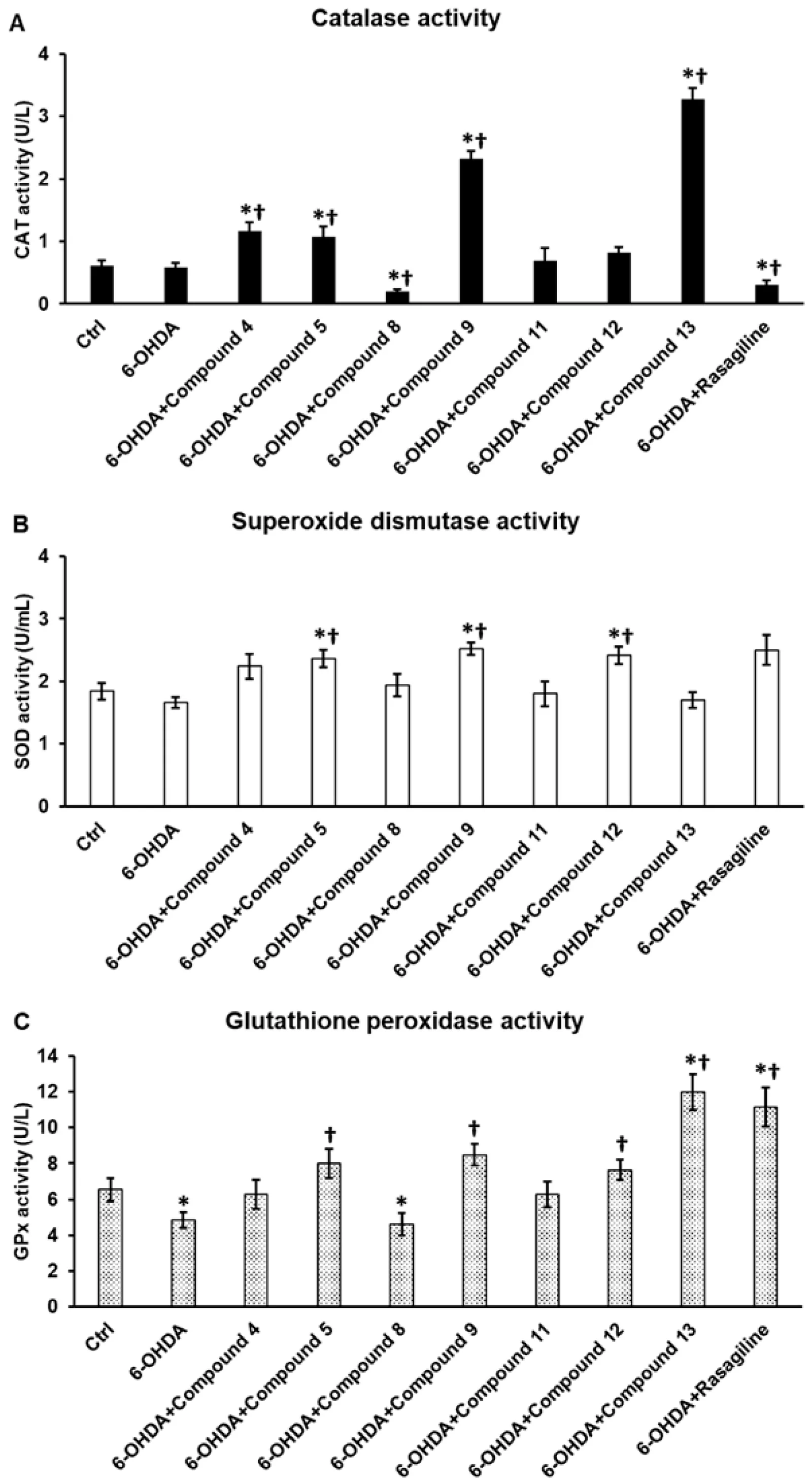
Activity measurements of three antioxidant enzymes in co-cultured differentiated SH-SY5Y cells. After treatment and cell separation, enzyme activities were determined using Catalase Assay (**A**), Superoxide Dismutase Assay (**B**), and Glutathione Peroxidase Assay (**C**) Kits. Cat and GPx activities were expressed as U/L, and SOD activity was expressed as U/mL. The columns represent the mean values ± standard deviation of three independent experiments. The asterisk indicates *p* < 0.05 compared to the control, and the cross represents *p* < 0.05 compared to the 6-OHDA treatment.

6-OHDA did not affect antioxidant enzyme activity, except for GPx, which was reduced in the SH-SY-5Y cells (Figure 10C). Compounds **5** (GPx, *p* = 0.0041; SOD, *p* = 0.0018; Cat, *p* < 0.0084) and **9** (GPx, *p* = 0.0012; SOD, *p* = 0.0003; Cat, *p* < 0.0001) significantly induced the activity of all three enzymes compared to 6-OHDA treatment (Figure 10), suggesting their potent antioxidant effects. Compound **12** significantly increased SOD (*p* = 0.0013) and GPx (*p* = 0.0026) activities (Figure 10B,C), whereas compound **13** significantly elevated catalase (*p* < 0.0001) and GPx (*p* = 0.0003) activities (Figure 10A,C). Compound **4** significantly increased catalase activities (*p* = 0.0041), whereas compound **8** reduced them (*p* = 0.0012) (Figure 10A). Compound **11** was ineffective against antioxidant enzymes.

Based on these results, curcuminoid derivatives and chalcones appear to alter the action of antioxidant enzymes differently. Therefore, they contribute to antioxidant defense at different ratios.

### 3.6. Cyclic C_5_-Curcuminoids and Chalcones Decrease the Iron-Mediated Oxidative Stress and the Markers of Ferroptosis in the Co-Cultured SH-SY5Y Cells

**Figure 11:**
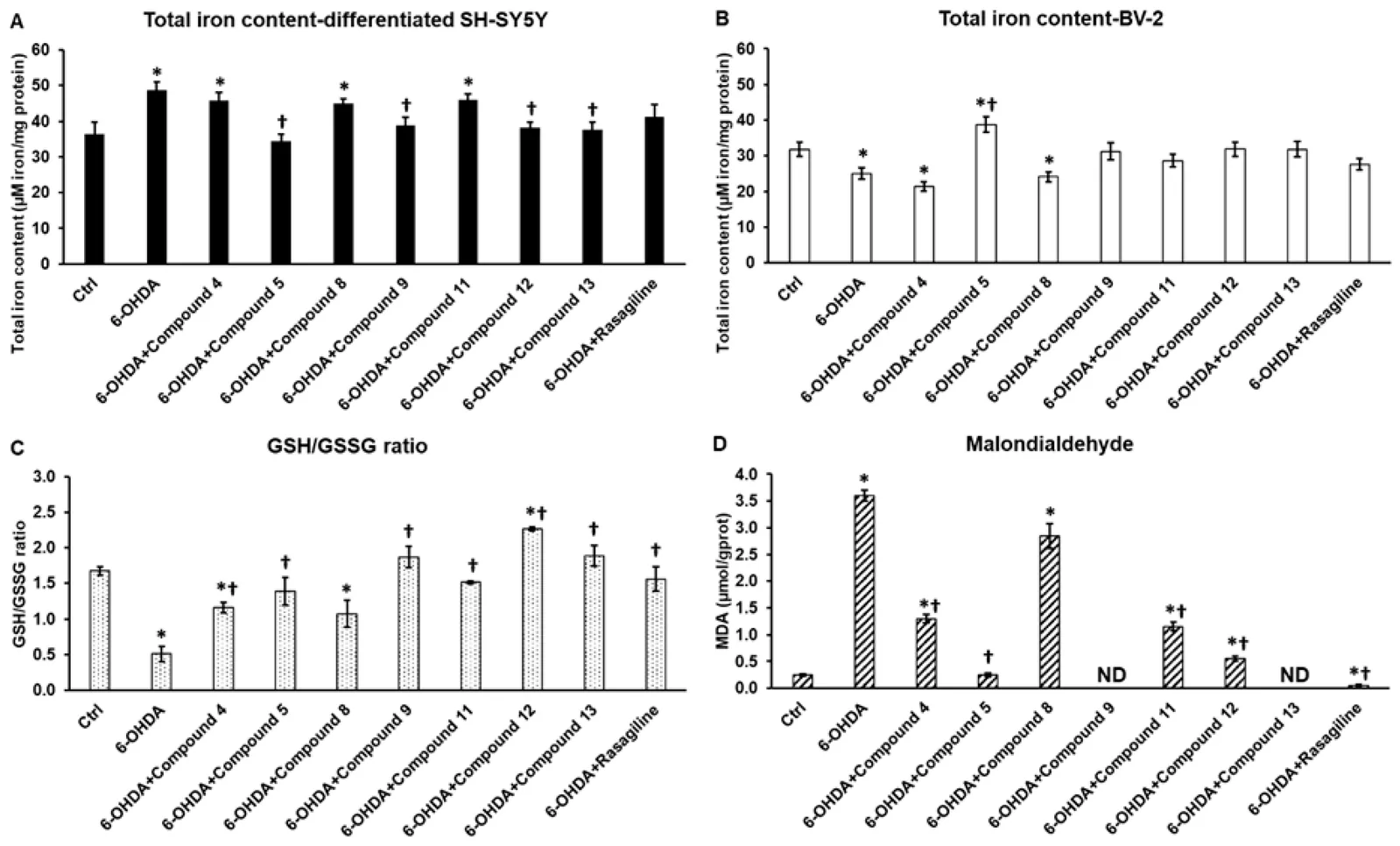
Measurements of total iron content (**A**,**B**), glutathione (**C**), and malondialdehyde (**D**) as markers of ferroptosis. After treatment and cell separation, the intracellular iron content of both cell types was determined using a ferrozine-based colorimetric method and was expressed as µM iron/mg protein. The reduced/oxidized glutathione ratio was determined using a GSH/GSSG Assay Kit. The malondialdehyde levels in the co-cultured SH-SY5Y cells were determined using the Malondialdehyde Colorimetric Assay Kit and expressed in µmol/g protein. The columns represent the mean values ± standard deviation of three independent experiments. The asterisk indicates *p* < 0.05 compared to the control, and the cross represents *p* < 0.05 compared to the 6-OHDA treatment. Abbreviations: GSH, reduced glutathione; GSSG, oxidized glutathione; ND, not detected.

The total iron content of the co-cultured SH-SY5Y cells significantly increased upon 6-OHDA treatment, while in the co-cultured BV-2 cells, reduced iron levels were detected (Figure 11A,B), suggesting iron transport between the two cell types. Compounds **5** (*p* = 0.001), **9** (*p* = 0.0048), **12** (*p* = 0.0023), and **13** (*p* = 0.0028) lowered iron levels in neurons compared to 6-OHDA treatment (Figure 11A). Interestingly, compound **5** (*p* = 0.0009) significantly increased the iron levels in the co-cultured BV-2 cells (Figure 11B).

The GSH/GSSG ratio indicates the severity of oxidative stress. The GSH/GSSG ratio demonstrated that 6-OHDA treatment increased oxidative stress. Meanwhile, compounds **5** (*p* = 0.0023), **9** (*p* = 0.0002), **12** (*p* < 0.0001), and **13** (*p* = 0.0002) decreased oxidative stress compared to 6-OHDA treatment in co-cultured SH-SY5Y cells and reached or exceeded control levels (Figure 11C). Compounds **4** (*p* = 0.001), **8** (*p* = 0.01), and **11** (*p* < 0.0001) also reduced oxidative stress but did not reach the control GSH/GSSG ratios (Figure 11C).

Malondialdehyde (MDA), a marker of lipid peroxidation, was significantly increased in the 6-OHDA-treated co-cultured SH-SY5Y cells (*p* < 0.0001) (Figure 11D). All curcuminoids and chalcones, except compound **8** (with the lowest GSH/GSSG ratio), significantly reduced MDA levels compared to 6-OHDA treatment. Compound **5** reduced the MDA levels to the control level, while MDA levels were below the detection limit in the case of cells treated with compounds **9** and **13** (Figure 11D).

### 3.7. Cyclic C_5_-Curcuminoids and Cyclic Chalcones Inhibit Apoptosis in the Co-Cultured SH-SY5Y Cells

**Figure 12:**
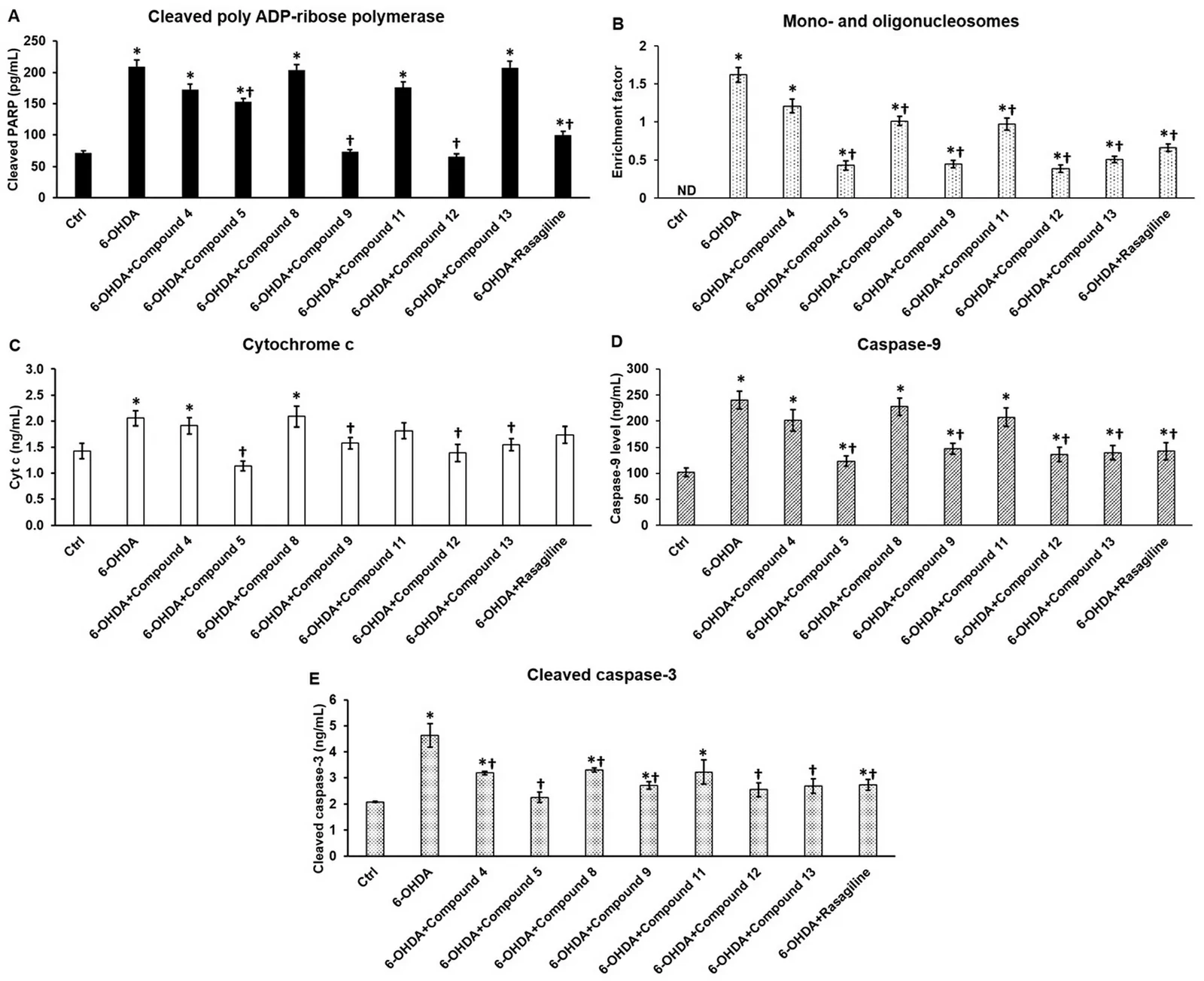
Concentrations of cleaved poly ADP-ribose polymerase (**A**), mono- and oligonucleosomes (**B**), cytochrome c (**C**), caspase-9 (**D**), and cleaved caspase-3 (**E**) in co-cultured differentiated SH-SY5Y cells after treatment with synthetic cyclic C_5_-curcuminoids. After treatment and cell separation, the intracellular concentrations of cleaved PARP, histone-associated DNA fragments, Cyt c, caspase-9, and cleaved Cas-3 were determined using Human PARP (Cleaved) [214/215], the Cell Death Detection ELISA^PLUS^, the Human Cytochrome C ELISA Kit, the Human Caspase 9 ELISA Kit, and the Human Caspase 3 (Cleaved) ELISA Kits. The columns represent the mean values ± standard deviation of three independent experiments. The asterisk indicates *p* < 0.05 compared to the control, and the cross represents *p* < 0.05 compared to the 6-OHDA treatment.

The concentration of cleaved (inactive) PARP significantly increased in the 6-OHDA-treated co-cultured SH-SY5Y cells (*p* < 0.0001) (Figure 12A). Compounds **5** (*p* = 0.001), **9** (*p* < 0.0001), and **12** (*p* < 0.0001) significantly reduced cleaved PARP levels compared to 6-OHDA treatment. Moreover, compounds 9 and **12** reduced the cleaved PARP concentrations to the control level (Figure 12A), suggesting strong inhibition of DNA fragmentation.

The release of histone-associated DNA fragments (mono- and oligonucleosomes) was also monitored to follow DNA damage in differentiated SH-SY5Y cells. 6-OHDA treatment of co-cultured SH-SY5Y cells significantly increased the enrichment factor of oligonucleosomes, suggesting DNA fragmentation by nucleases activated by the intrinsic apoptotic pathway. Compounds **5** (*p* < 0.0001), **9** (*p* < 0.0001), **12** (*p* < 0.0001), and **13** (*p* < 0.0001) decreased the enrichment factor of histone-associated DNA fragments, suggesting the inhibition of endonucleases and DNA fragmentation (Figure 12B) and apoptosis.

6-OHDA treatment of co-cultured SH-SY5Y cells significantly increased cytochrome c levels compared to the control (*p* = 0.0062) (Figure 12B). Compounds **5** (*p* = 0.0007), **9** (*p* = 0.0106), and **12** (*p* = 0.0067) significantly reduced cytochrome c concentrations compared to 6-OHDA. Compound **5** significantly reduced cytochrome c levels; compound 13 did not (Figure 12C).

Because the total cytochrome c level alone does not prove the activation of apoptosis, the active caspase-9 levels were also measured. 6-OHDA treatment of co-cultured SH-SY5Y cells significantly increased caspase-9 levels (*p* = 0.0002), whereas compounds **5** (*p* = 0.0005), **9** (*p* = 0.0013), **12** (*p* = 0.0012), and **13** (*p* = 0.014) significantly reduced them (Figure 12D).

In the case of cleaved caspase-3 (effector caspase), increased levels were observed in the 6-OHDA-treated co-cultured SH-SY5Y cells (Figure 12E). Compounds **5** (*p* = 0.0011), **9** (*p* = 0.0022), **12** (*p* = 0.0024), and **13** (*p* = 0.0031) reduced the levels of cleaved caspase-3 (Figure 12E). Compounds **4** (*p* = 0.0054) and **8** (*p* = 0.0074) also reduced cleaved caspase-3, though the effect was less convincing.

These results suggest that compounds **5**, **9**, and **12** possess high anti-apoptotic potential in the 6-OHDA-induced neuronal damage model. Compound **13** also inhibited DNA fragmentation and the levels of active caspase-9 and -3. These results suggest that compound **13** may increase the production of inhibitors of apoptosis (IAPs).

### 3.8. Cyclic C_5_-Curcuminoids and Cyclic Chalcones Modify the Intracellular and Extracellular Glutamate Levels in the Co-Cultured Cells

**Figure 13:**
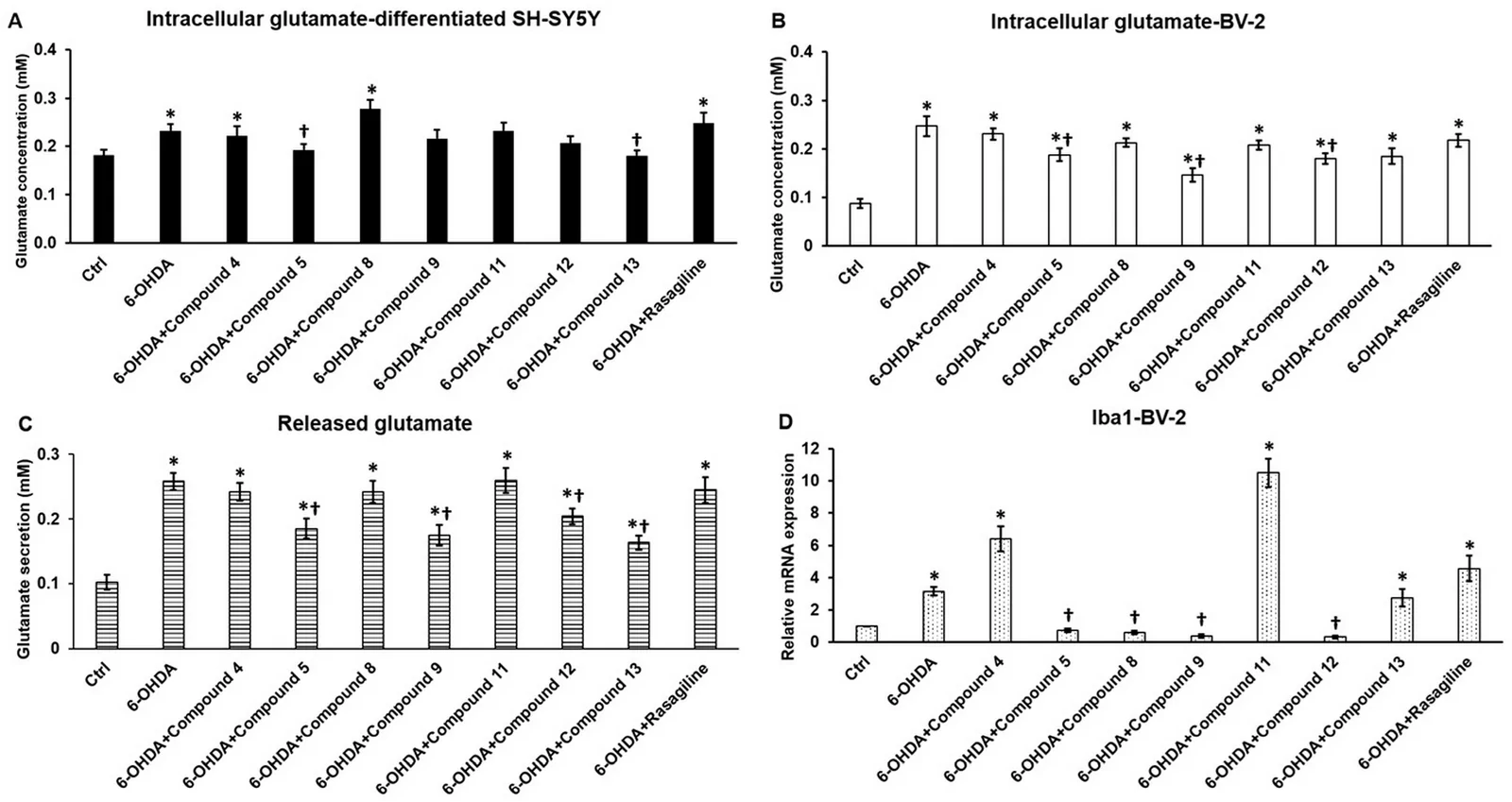
Determination of intracellular glutamate content in co-cultured cells (**A**,**B**), extracellular glutamate content (**C**), and Iba1 mRNA expression (**D**) in BV-2 cells. After treatment and cell separation, the intracellular glutamate concentrations were measured using the Glutamate Assay-Kit-WST and expressed in mM. Iba1 expression was determined using an SYBR Green-based real-time PCR protocol. Target gene expression was normalized to β-actin, a housekeeping gene, and compared with the control level. The columns represent the mean values ± standard deviation of three independent experiments. The asterisk indicates *p* < 0.05 compared to the control, and the cross represents *p* < 0.05 compared to the 6-OHDA treatment. Abbreviation: Iba1, ionized calcium-binding adapter molecule 1.

Interestingly, similar results were observed in both differentiated SH-SY5Y cells and BV-2 microglia upon 6-OHDA treatment; glutamate levels were significantly elevated (SH-SY5Y *p* = 0.0075; BV-2 *p* < 0.0001) (Figure 13A–C).

Only compounds **5** (*p* = 0.02) and **13** (*p* = 0.0074) reduced intracellular glutamate levels in SH-SY5Y cells (Figure 13A). In contrast, in BV-2 cells, compounds **5** (*p* = 0.0142), **9** (*p* = 0.0022), and **12** (*p* = 0.0079) decreased glutamate levels (Figure 13B). Extracellular glutamate, although it did not reach excitotoxic levels, can exacerbate oxidative stress in differentiated SH-SY5Y cells. Compounds **5** (*p* = 0.0031), **9** (*p* = 0.0022), **12** (*p* = 0.0061), and **13** (*p* = 0.0006) significantly decreased glutamate release compared to the 6-OHDA treatments (Figure 13C).

*Iba1* mRNA level upregulation occurs shortly after initiating microglial activation. Interestingly, in addition to 6-OHDA, compounds **4** (*p* = 0.0003), **11** (*p* = 0.0001), **13** (*p* = 0.003) and rasagiline (*p* = 0.002) increased *Iba1* mRNA expression in microglia compared to control (Figure 13D). Compounds **5** (*p* = 0.0001), **8** (*p* = 0.0001), **9** (*p* < 0.0001), and **12** (*p* < 0.0001) significantly decreased *Iba1* mRNA levels compared to 6-OHDA treatment (Figure 13D).

Based on these results, compounds **5**, **9** (although it did not influence SH-SY5Y glutamate levels), **12**, and **13** are promising candidates for reducing microglia-driven oxidative stress and neuronal damage.

### 3.9. Cyclic C_5_-Curcuminoids 9, 12, and 13 Increase the Secretion of IL-10 Anti-Inflammatory Cytokine

**Figure 14:**
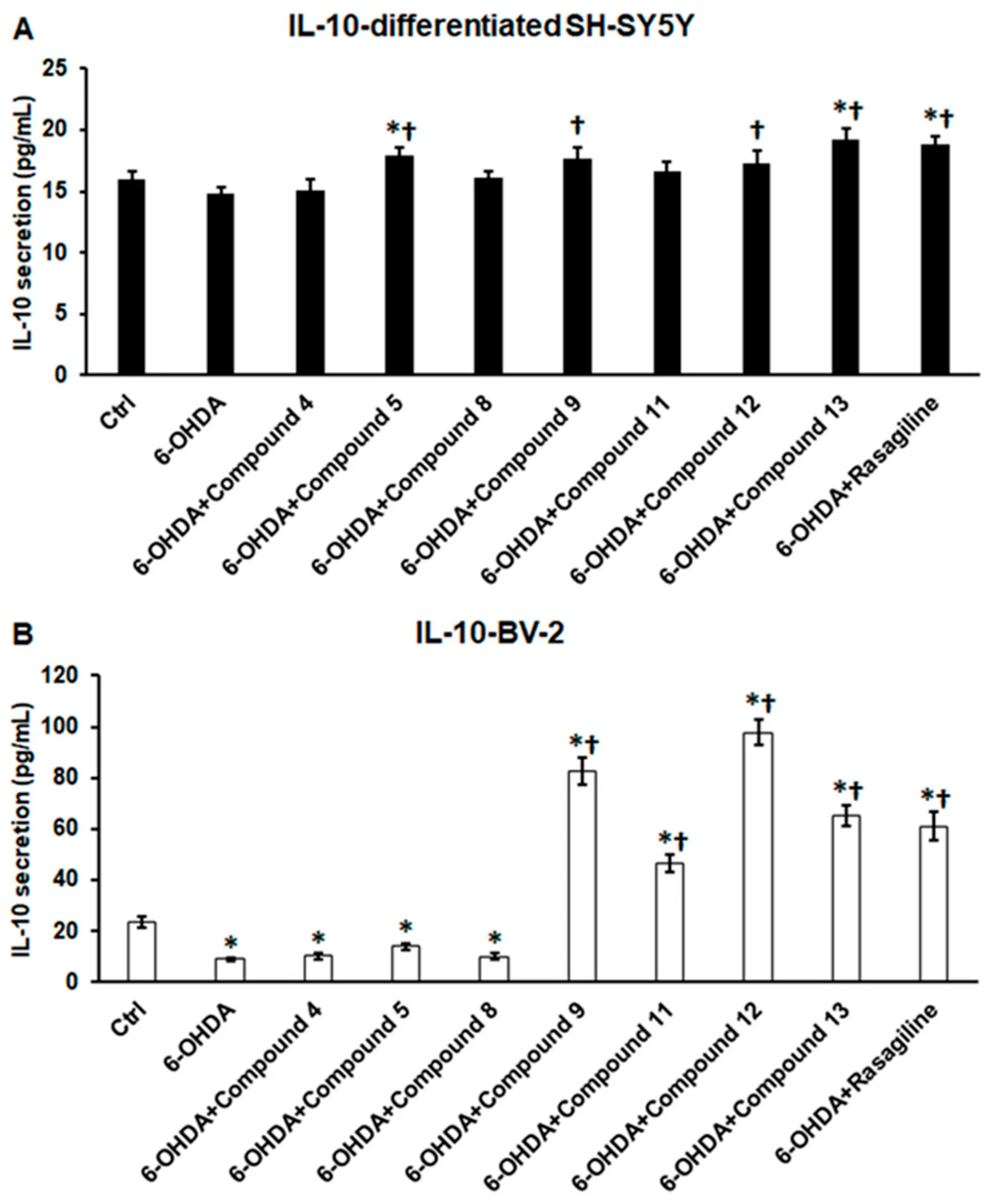
Concentration measurements of IL-10 anti-inflammatory cytokine secreted by neurons or microglia. After treatment, IL-10 levels were determined from the cell culture supernatants using the Human IL-10 ELISA kit for SH-SY5Y cells (**A**) and the Mouse IL-10 ELISA Kit for BV-2 cells (**B**). IL-10 concentrations were expressed in pg/mL. The columns represent the mean values ± standard deviation of three independent experiments. The asterisk indicates *p* < 0.05 compared to the control, and the cross represents *p* < 0.05 compared to the 6-OHDA treatment.

In our study, 6-OHDA treatment did not affect neuronal IL-10 secretion but decreased it in BV-2 cells in co-cultures (Figure 14A). Compounds **5** (*p* = 0.004), **9** (*p* = 0.0091), **12** (*p* = 0.022), and **13** (*p* = 0.0022), as well as rasagiline (*p* = 0.0013), significantly increased IL-10 secretion in SH-SY5Y cells compared to 6-OHDA treatment (Figure 14A). In BV-2 microglia, compounds **4** (*p* = 0.0006), **5** (*p* = 0.0021), and **8** (*p* = 0.0007) significantly reduced IL-10 secretion compared to that of the control (Figure 14B). In contrast, compounds **9** (*p* < 0.0001; *p* < 0.0001), **11** (*p* = 0.0005; *p* < 0.0001), **12** (*p* < 0.0001; *p* < 0.0001), and **13** (*p* = 0.0001; *p* < 0.0001) significantly elevated IL-10 production in BV-2 cells compared to both 6-OHDA treatment and control (Figure 14B). For compound **5**, a different mechanism of action can be hypothesized: in microglia, the reduced IL-10 level tends to maintain a pro-inflammatory state, whereas in neurons, it increases IL-10 production, potentially inhibiting the production of inflammatory cytokines. This may be due to differences in the regulation of cytokine-regulating signaling pathways.

### 3.10. Cyclic C_5_-Curcuminoids and Cyclic Chalcones Reduce the Secretion of the Pro-Inflammatory Cytokines IL-6 and TNFα

**Figure 15:**
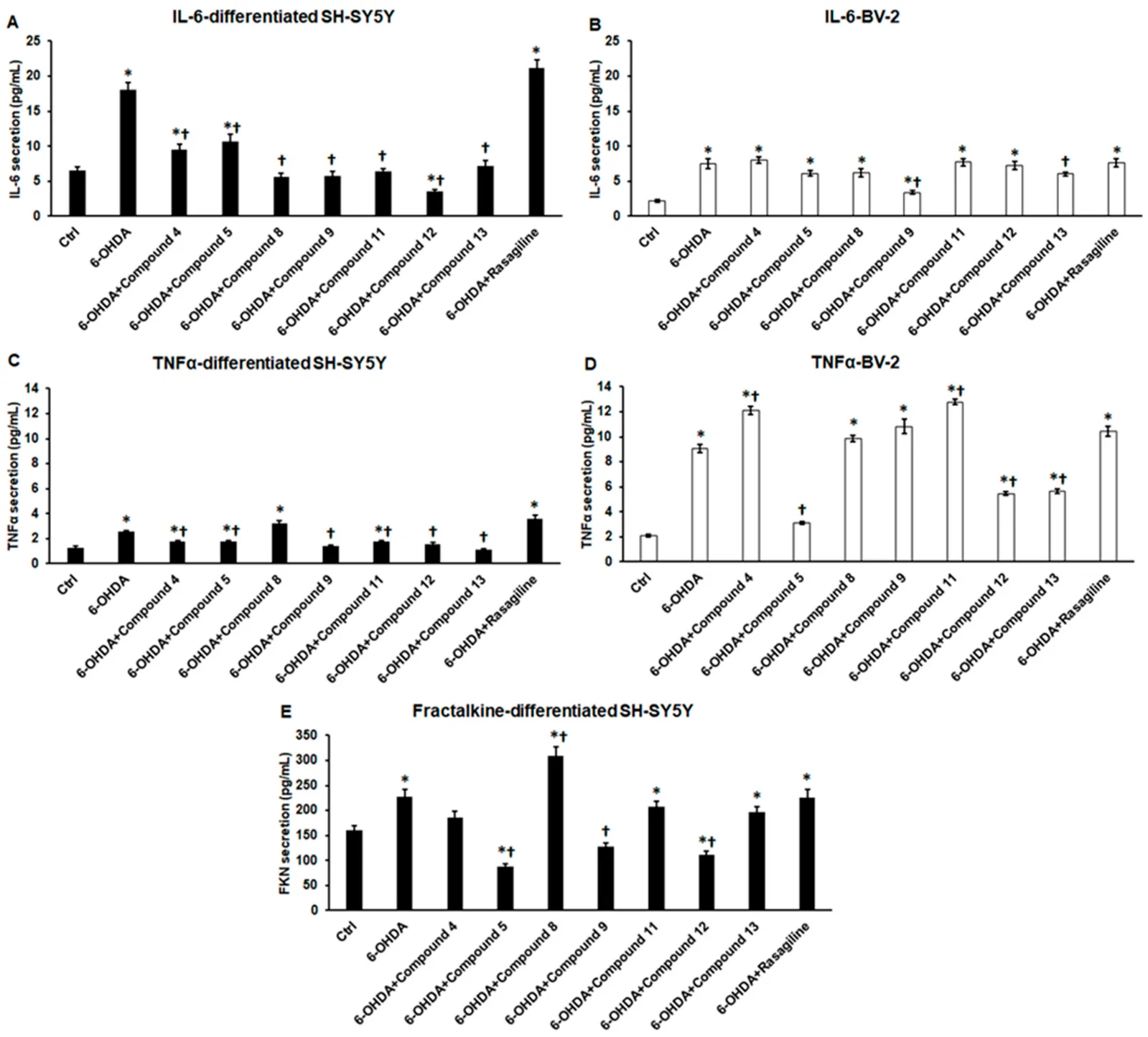
Concentration measurements of IL-6 and tumor necrosis factor α, pro-inflammatory cytokines secreted by neurons (**A**,**C**) and microglia (**B**,**D**), and concentration measurements of fractalkine chemokine secreted by co-cultured SH-SY5Y cells (**E**). After treatment, cytokine and chemokine levels were determined from the cell culture supernatants using the Human IL-6 and Human TNFα ELISA and Human Fractalkine ELISA Kits for SH-SY5Y cells and the Mouse IL-6 and Mouse TNFα ELISA Kits for BV-2 cells. Cytokine and chemokine concentrations were expressed in pg/mL. The columns represent the mean values ± standard deviation of three independent experiments. The asterisk indicates *p* < 0.05 compared to the control, and the cross represents *p* < 0.05 compared to the 6-OHDA treatment. Abbreviations: 6-OHDA, 6-hydroxydopamine; IL-6, interleukin-6; FKN, fractalkine; TNFα, tumor necrosis factor α.

6-OHDA treatment significantly increased IL-6 and TNFα pro-inflammatory cytokine secretion in both cell types in the co-cultures. It should be noted that SH-SY5Y cells released more IL-6, whereas BV-2 cells secreted more TNFα pro-inflammatory cytokines into the culture medium (Figure 15). All compounds reduced neuronal IL-6 secretion (Figure 15A). In BV-2 cells, only compounds **9** (*p* = 0.0008) and **13** (*p* = 0.0349) decreased IL-6 release compared to 6-OHDA treatment (Figure 15B).

In the case of TNFα, all compounds except compound **8** decreased TNFα secretion in SH-SY5Y cells (Figure 15C). In BV-2 microglia, compounds **5** (*p* < 0.0001), **12** (*p* = 0.0001), and **13** (*p* = 0.0001) reduced TNFα release compared to 6-OHDA treatment (Figure 15D).

Based on the results, we can conclude that only compound **13** is capable of reducing IL-6 and TNFα secretion in both cell types in the co-culture, although compounds **9** and **12** are also promising anti-inflammatory molecules. The mechanism of action of compound 5 appears to vary between the two cell types. In neurons, it decreased the production of both IL-6 and TNFα, whereas in microglia, it only reduced the secretion of TNFα. This discrepancy may result from differences in the regulation of the signaling pathways.

6-OHDA treatment significantly increased fractalkine secretion in co-cultured differentiated SH-SY5Y cells (*p* = 0.0023) (Figure 15E). Interestingly, compound **8** (*p* = 0.0038) elevated the fractalkine level after 6-OHDA treatment, suggesting its microglial-activating effect (Figure 15E). Compounds **5** (*p* < 0.0001), **9** (*p* = 0.0005), and **12** (*p* = 0.0003) significantly reduced the release of fractalkine compared to 6-OHDA treatment (Figure 15E). Interestingly, compound **13**, which reduced the levels of the pro-inflammatory cytokines IL-6 and TNFα, did not decrease fractalkine secretion.

### 3.11. Cyclic C_5_-Curcuminoids and Cyclic Chalcones Alter the Expression of p105, p50, and Phospho-p65, Regulating Inflammation in Co-Cultured SH-SY5Y Cells

**Figure 16:**
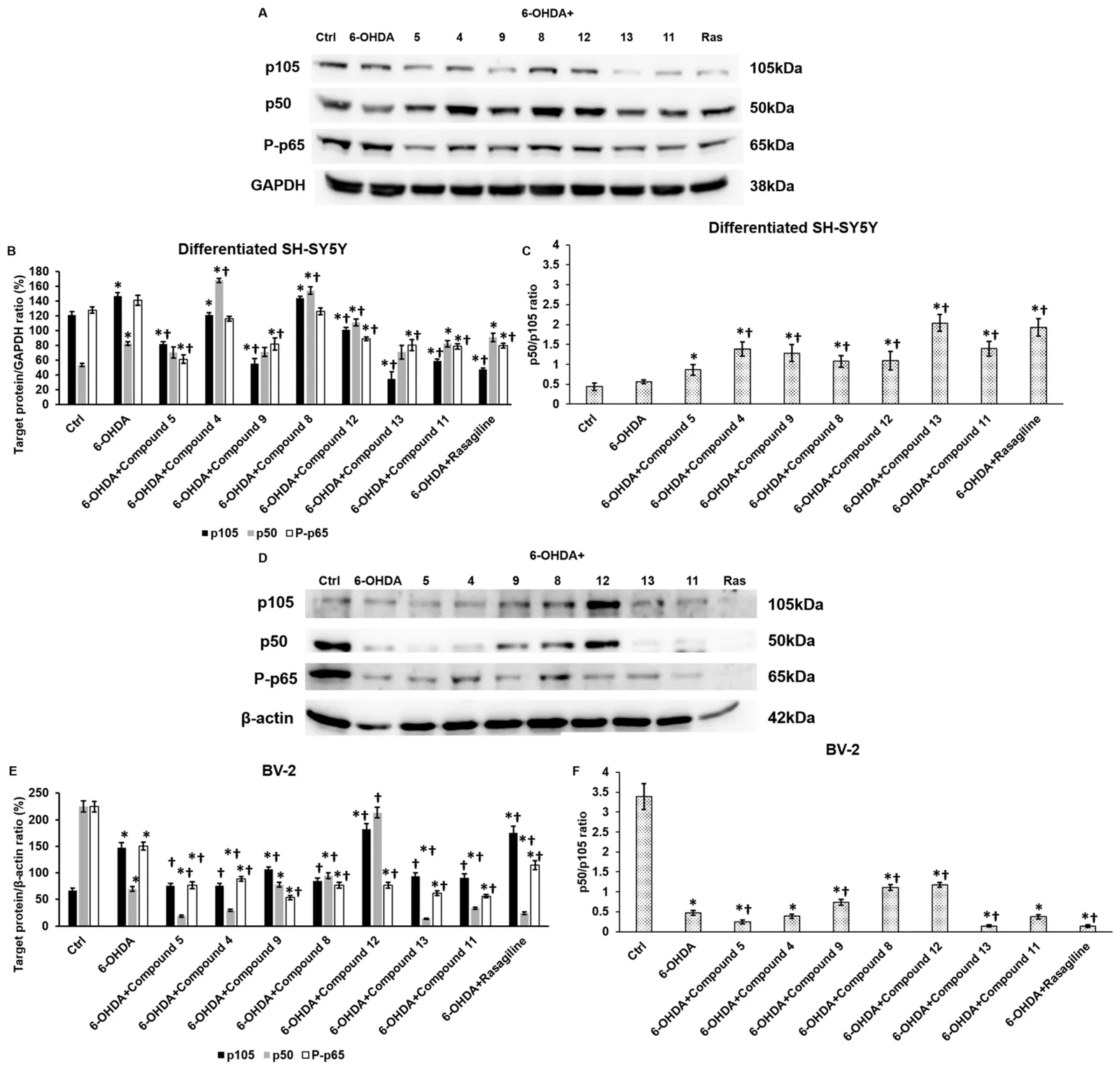
Western blot analysis of NFκB1 p105, p50, and phospho-p65 in co-cultured SH-SY5Y cells and BV-2 cells after curcuminoid treatment. After treatment and cell separation, the cell pellets were lysed, protein content was measured, and an equal amount of protein was separated on SDS–polyacrylamide gels. After blotting, the membranes were probed with anti-p105/p50 and anti-phospho-p65 antibodies. GAPDH was used as the loading control for SH-SY5Y cells and β-actin for BV-2 cells. Target protein expression was expressed as a percentage of the target/GAPDH ratio. (**A**) Representative Western blots of target proteins and loading controls of SH-SY5Y cells. (**B**) Analysis of protein expression in SH-SY5Y cells. (**C**) p50/p105 protein ratio in differentiated SH-SY5Y cells. (**D**) Representative Western blots of target proteins and loading controls of BV-2 cells. (**E**) Analysis of protein expression in BV-2 cells. (**F**) p50/p105 protein ratio in BV-2 cells. The columns represent the mean values ± standard deviation. The asterisk indicates *p* < 0.05 compared to the control, and the cross represents *p* < 0.05 compared to the 6-OHDA treatment. Abbreviations: GAPDH, glyceraldehyde 3-phosphate dehydrogenase.

6-OHDA treatment significantly increased p105 and p50 protein levels in co-cultured SH-SY5Y cells but only slightly increased P-p65 levels (Figure 16A,B). Compounds **5** (*p* = 0.0003), **9** (*p* = 0.0002), **11** (*p* = 0.0002), **12** (*p* = 0.0005), and **13** (*p* = 0.0001) significantly reduced p105 levels compared to 6-OHDA treatment (Figure 16B); however, none of the compounds decreased p50 levels. Moreover, compounds 4 and 8 further increased p50 levels compared to 6-OHDA treatment (Figure 16A,B). The elevation in the p50/p105 protein ratio indicates the activation of the signaling pathway (Figure 16C). In contrast, all compounds, except compounds **4** and **8**, significantly reduced P-p65 protein levels compared to 6-OHDA and the controls (Figure 16A,B), suggesting that the anti-inflammatory effects of cyclic C_5_-curcuminoids and cyclic chalcones are based on the inhibition of p65 phosphorylation in SH-SY5Y cells.

In BV-2 cells, 6-OHDA significantly increased the protein levels of p105 (*p* = 0.0002) but decreased p50 (*p* < 0.0001) and P-p65 (*p* = 0.0005) levels (Figure 16D,E). Compounds **4** (*p* = 0.0002), **5** (*p* = 0.0002), **9** (*p* = 0.0022), **11** (*p* < 0.0011), and **13** (*p* = 0.0011) significantly reduced p105 levels compared to 6-OHDA treatment alone (Figure 16E). However, compounds **4** (*p* = 0.0001), **5** (*p* < 0.0001), **11** (*p* = 0.0002), and **13** (*p* < 0.0001) decreased the p50 levels, too (Figure 16D,E). The p50/p105 ratio significantly decreased only after treatment with compounds **5** (*p* = 0.007) and **13** (*p* = 0.001) compared to 6-OHDA treatment (Figure 16F).

All compounds significantly lowered P-p65 protein levels compared to 6-OHDA and the controls (Figure 16D,E). These results suggest that compounds **5** and **13** exert anti-inflammatory effects in BV-2 cells. However, the other compounds may also possess anti-inflammatory effects by inhibiting p65 phosphorylation.

## 4. Discussion

Neurodegenerative disorders such as Parkinson’s disease, Alzheimer’s disease, Huntington’s disease, and amyotrophic lateral sclerosis are prevalent worldwide [43]. Common features include protein aggregation, oxidative stress, disrupted iron metabolism, ferroptosis, mitochondrial dysfunction, and microglial activation, which drive disease progression [44,45,46]. Microglia protect the brain by monitoring functions, removing materials and damaged cells, supporting neurons, and regulating neuroinflammation through cytokines and chemokines [28,47]. However, chronic activation harms neurons and increases neuronal death. In neurodegenerative diseases, active microglia promote ROS production, oxidative stress, and excess glutamate release, leading to increased cell death [28,48]. Thus, inhibiting microglial activation is a therapeutic issue, while treatments relieve symptoms and slow the disease progression.

Natural, bioactive compounds found in plants are under massive investigation to treat neurodegenerative diseases [49,50,51], including BBB protection [13]. Although many natural substances have shown promising results (e.g., curcumin, resveratrol, linalool, geraniol, and limonene), there are some challenges regarding their bioavailability, stability, and ability to cross the blood–brain barrier [52,53,54].

Curcumin is extracted from turmeric (*Curcuma longa* L., *Curcuma domestica* L.) [3]. It has been described as an antioxidant and anti-inflammatory molecule [55,56]. It decreased activation of the nuclear factor kappa-light-chain-enhancer of activated B cells (NFκB) and Janus kinase/Signal transducer and activator of transcription (JAK/STAT) signaling pathways and reduced synthesis of pro-inflammatory cytokines such as IL-6, IL-1β, and TNFα [57]. In preclinical studies, inhibition of the NFκB pathway and decreased pro-inflammatory cytokines were central to the BBB-protective effects of curcumin [13]. The major problems associated with curcumin are its hydrophobic nature, low stability, and rapid metabolism [58]. Stability and biological activity have been improved using metal complexes, nanoparticles, and more stable synthetic curcuminoid analogs, including synthetic C_5_-curcuminoids as neuroprotective agents in mice (including in vitro and in vivo tests and molecular docking validation) and cyclic C_5_-curcuminoids [15,20].

In our previous study, we examined seven synthetic cyclic C_5_-curcuminoids and chalcones in monocultures of all-trans retinoic acid-differentiated SH-SY5Y cells. Three cyclic C_5_-curcuminoids and one chalcone showed potential antioxidant and anti-apoptotic effects, but their anti-inflammatory effects remain incompletely investigated [26].

In the present study, we developed an in vitro neuronal damage co-culture model using differentiated SH-SY5Y cells, BV-2 microglia, and 6-OHDA to mimic oxidative stress in SH-SY5Y cells and monitor the effects of microglia on neurons. Therefore, it was possible to prove the advantageous effects of the synthetic cyclic C_5_-curcuminoids and chalcones even in the presence of microglia, which, in an active state, can contribute to neuronal loss [28,48].

6-OHDA treatment enhances ROS production, contributing to oxidative stress [59]. It also triggers iron accumulation by altering transferrin receptor 1 and ferroportin expression [60,61]. Increased labile iron activates the Fenton reaction, whereas 6-OHDA auto-oxidation generates superoxide anions and hydrogen peroxide [62]. Mitochondrial respiratory chain inhibition further increases superoxide production, causing mitochondrial damage and reducing energy production [63]. Antioxidant enzymes eliminate reactive oxygen radicals in compartments [64]. GPx protects membranes from lipid peroxidation and ferroptosis [65], SOD protects against superoxides, and catalase eliminates hydrogen peroxide [66,67]. Regarding antioxidant protection, ROS and TAC levels and GPx and SOD activities correlated with measurements in SH-SY5Y monocultures from our publication [26]. However, BV-2 microglia in co-cultures altered compound effectiveness. In co-cultures, compounds **5**, **9**, **12**, and **13** enhanced protection of neurons via ROS, TAC, GPx, and catalase measurements with values lower than those observed in monocultures [26]. Compounds **5** and **9** increased the activities of all three antioxidant enzymes, suggesting that synthetic C_5_-curcuminoids affect defense mechanisms. Based on the GSH/GSSG ratio, all tested compounds reduced oxidative stress compared to 6-OHDA in differentiated SH-SY5Y cells, but only compounds **5**, **9**, **12**, and **13** restored control levels, possibly through GSH synthesis and GPx-GR activity [68].

Elevated intracellular iron content, especially in the labile iron pool containing reactive ferrous iron, can strongly contribute to iron-mediated oxidative stress, affecting glutathione levels and GPx activity [69]. In neurodegeneration, microglia may also contribute to neuronal iron accumulation by releasing iron and triggering its uptake into the neurons, which may enhance lipid peroxidation, leading to ferroptosis [70,71,72]. In co-cultures, 6-OHDA-treated SH-SY5Y cells exhibited increased iron levels, supporting 6-OHDA-induced iron accumulation. Compounds **5**, **9**, **12**, and **13** maintained control iron levels in neurons after 6-OHDA exposure, protecting against iron-mediated ROS production. In BV-2 cells, compounds **9**, **12**, and **13** did not alter iron levels, whereas compound **5** increased microglial iron. These results indicate that BV-2 cells likely alter the expression of genes involved in iron uptake and release [40].

Malondialdehyde level, which is a potential marker for oxidative stress and an indicator of lipid peroxidation [73], significantly decreased in the differentiated SH-SY5Y cells using three cyclic C_5_-curcuminoids (**9**, **12**, and **13**) and one chalcone (**5**), suggesting balanced oxidative protection and reduced oxidants.

Apoptosis contributes to neuronal loss in neurodegenerative diseases [74]. DNA damage in Parkinson’s disease activates PARP-1, responsible for DNA repair. During apoptosis, PARP-1 is cleaved into 89 and 24 kDa fragments, leading to the inactivation of the repair enzyme. Consequently, DNA fragmentation and cell death occur [75]. In our experiments, 6-OHDA treatment significantly increased the concentration of cleaved PARP in differentiated SH-SY5Y cells in co-cultures, whereas compounds **5, 9**, and **12** reversed the effect of 6-OHDA, suggesting a decrease in DNA damage. Interestingly, compound **13**, which has a strong antioxidant defense-activating effect, did not reverse the effect of 6-OHDA on PARP cleavage. The inhibition of DNA fragmentation was proven by the significantly decreased level of mono- and oligonuceosomes in the cytosol of the SH-SY5Y cells upon treatment with compounds **5**, **9**, **12**, and **13**.

Oxidative stress-mediated mitochondrial dysfunction initiates the release of cytochrome c from the mitochondria, activating the caspase cascade. At the end of programmed cell death, the final caspase, executioner caspase-3, is activated [76,77]. Total cytochrome c levels decreased in the co-cultured SH-SY5Y cells treated with compounds **5**, **9**, **12**, and **13**. However, this phenomenon did not prove the activation of the mitochondrial apoptotic pathway. Caspase-9, activated by the apoptosome, and cleaved caspase-3 levels support the apoptosis-inhibitory effects of compounds **5**, **9**, **12**, and **13**. This may be due to decreased oxidative damage to the mitochondria. Interestingly, in the case of caspase-3 activation, not only the aforementioned four compounds but also compounds **4** and **8** successfully decreased the level of active caspase-3. One possible explanation for this is that these compounds may increase the expression of inhibitors of apoptosis (IAPs) [78].

Microglia expressing ionized calcium-binding adapter molecule (Iba1) activation marker secrete pro-inflammatory cytokines (TNFα, IL-6, IL-1), driving neuroinflammation [79,80]. Furthermore, oxidative stress in the brain or the release of pro-inflammatory cytokines from neurons rapidly upregulates Iba1 expression, and microglia turn to an active state [81], exacerbating oxidative stress and inflammation, and contributing to neuronal damage [33].

The expression of Iba1 mRNA increases shortly after the onset of activation; however, this alteration alone does not confirm microglial activation. It has been revealed that 6-OHDA treatment upregulated Iba1 expression in co-cultured BV-2 cells, whereas compounds **5**, **8**, **9**, and **12** significantly decreased the expression of this marker. Together with the elevated secretion of pro-inflammatory cytokines, specifically TNFα and IL-6, by microglia in a co-culture with 6-OHDA-pretreated differentiated SH-SY5Y cells, this indicates the development of an inflammatory phenotype. The production of inflammatory cytokines requires enhanced activity of the signaling pathways associated with their activation.

Activated microglia release glutamate, an excitotoxin, exacerbating neuronal loss by activating receptors and causing Ca homeostasis disruption, ROS production, mitochondrial dysfunction, and inflammation [82,83]. In neurodegeneration, neuronal glutamate activates microglial glutamatergic receptors, aggravating neuroinflammation and further impairing antioxidant defense [83,84,85].

Interestingly, glutamate production in BV-2 cells decreased only with compounds **5**, **9**, and **12**. In differentiated co-cultured SH-SY5Y cells, only compounds **5** and **13** effectively decreased glutamate production compared to 6-OHDA treatment. Intracellular glutamate levels do not underlie the glutamate-mediated excitotoxicity. The glutamate release was also measured in the culture medium of the co-cultures, showing decreasing levels in the case of compounds **5**, **9**, **12**, and **13**. However, in this case, it is not possible to separate human- and mouse-originating glutamate concentrations. In this model, we cannot achieve excitotoxic levels of glutamate, since BV-2 microglia can release around 200–300 µM glutamate. However, the elevated intracellular glutamate levels in BV-2 microglia and the 200–300 µM extracellular glutamate in the co-culture model can drive oxidative stress in SH-SY5Y cells [86,87]. Therefore, we cannot speak about classical receptor-mediated excitotoxicity, but it is hypothesized that glutamate modifies the activity of the cystine/glutamate antiporter (SLC17A). Consequently, it decreases GSH levels (decreased GSH/GSSG ratio), as well as the activity of glutathione peroxidase, which were both observed in the 6-OHDA pre-treated SH-SY5Y cells of the co-cultures.

Microglial activation is closely associated with neuroinflammation. In the brain, IL-10, an anti-inflammatory cytokine released by neurons, astrocytes, and microglia, contributes to maintaining the normal function and survival of the brain cells [88]. Downregulation of IL-10 promotes the activation of microglia towards the pro-inflammatory state [89]. Synthetic cyclic C_5_-curcuminoids (**9**, **12**, and **13**) and chalcone (**5**) significantly induced IL-10 secretion from co-cultured differentiated SH-SY5Y cells. However, compound 5 did not have the same effect on BV-2 microglia, suggesting a different mechanism of action for this chalcone in the regulation of cytokine synthesis. Compounds **9**, **11**, **12**, and **13** increased microglial IL-10 production, suggesting their anti-inflammatory roles in the 6-OHDA-induced neuronal damage model. For the pro-inflammatory cytokines TNFα and IL-6, all compounds decreased their levels (except for compound **4** for IL-6 and compound **8** for TNFα) in the neurons. In microglia, compounds **9** and **13** downregulated IL-6, whereas compounds **5**, **12**, and **13** reduced TNFα. Compound **13** decreased both pro-inflammatory cytokines in both cell types in the co-culture. The distinct effects of compound **5** on IL-6 and TNFα indicate that it may exert differential actions on neurons and microglia. This variation could be attributed to the distinct regulation of inflammatory pathways. In neurons treated with compound **5**, the JAK1/STAT3 pathway can be activated by IL-10, resulting in anti-inflammatory effects. Conversely, in microglia, the activation of JAK1/STAT3 is induced by IL-6, which subsequently promotes additional IL-6 synthesis [90,91].

Microglial activation and pro-inflammatory cytokine expression are regulated by fractalkine/fractalkine receptor (CX3CR1) interaction [92]. Soluble fractalkine, released from neuronal membrane-bound fractalkine, binds microglial receptors and upregulates the NFκB inflammatory pathway in a concentration-dependent manner and the mitogen-activated protein kinase (MAPK) pathway, regulating cell cycle, division, and apoptosis [93,94]. Soluble fractalkine contributes to neurodegenerative disease progression [95] and enhances neuronal iron accumulation, oxidative stress, and ferroptosis [96]. Its downregulation in co-cultured differentiated SH-SY5Y cells treated with compounds **5**, **9**, and **12** correlated with reduced pro-inflammatory cytokine expression, whereas compound **13** caused only a slight decrease in fractalkine secretion.

In the case of compound **13**, although IL-6 and TNFα secretion decreased, this was inconsistent with the increased expression of *Iba1* and the levels of fractalkine. Disruption of fractalkine signaling, such as the internalization of CX3CR1 on microglia due to increased soluble fractalkine levels or the loss of membrane-bound fractalkine on SH-SY5Y cells, can lead to microglial activation [92]. In the case of compound 13, the fractalkine level remained unchanged in comparison to 6-OHDA treatment, which may contribute to the increased expression of *Iba1* mRNA.

On the other hand, the discrepancy between fractalkine, *Iba1*, and pro-inflammatory cytokines may be due to the regulation of alternative signaling pathways: besides the major inflammatory pathway NFκB, other signaling pathways, such as C/EBPβ, MAPK/p38, JAK/STAT, and Akt [97,98], may also increase the expression of certain cytokines such as IL-6, IL-1β, IL-8, and TNFα [99].

Western blot analysis of NFκB transcription factors also supports this hypothesis. In the co-cultured differentiated SH-SY5Y cells, 6-OHDA treatment increased p105, p50, and P-p65 expression, which correlated with pro-inflammatory cytokine and fractalkine secretion. Compounds **5**, **9**, **11**, **12**, and **13** significantly reversed the effect of 6-OHDA by decreasing the expression of P-p65, which was observed in the alterations in pro-inflammatory cytokine production. In microglia, compounds **4**, **5**, **9**, **11**, and **13** significantly reduced the p105 protein levels and P-p65, whereas compounds **4**, **5**, **11**, and **13** decreased p50 levels. However, only compounds **5** and **13** significantly decreased the p50/p105 ratio, suggesting the downregulation of the signaling pathway. Based on these observations, synthetic cyclic C_5_-curcuminoids appear to mainly act by inhibiting p65 phosphorylation in SH-SY5Y cells, but in BV-2 cells they may also act via the downregulation of p50.

Concerning structure–activity relationship (SAR) analysis, we share the following points. This study selected compounds **4**, **5**, **8**, **9**, **11**, **12**, and **13** for testing as pairs with slight structural differences: phenolics (**5**, **9**, **12**, and **13**) and non-phenolics (**4**, **8**, and **11**), in which the phenolic function was blocked by a hardly removable methyl substituent. This distinction indicates a biochemical difference: in biological hydrogen transfer reactions, phenolics are hydrogen donor groups, whereas non-phenolic compounds **4, 8**, and **11** are neutral in this respect. Such enzyme-catalyzed hydrogen (hydride) transfer reactions are well known and ubiquitous in biological media [100,101]. The two groups could also be separated by biological efficacy, except for a few cases, throughout testing in this study, according to Section 3.3, Section 3.4, Section 3.5, Section 3.6, Section 3.7, Section 3.8, Section 3.9, Section 3.10 and Section 3.11 above. The hydrogen-donor phenolic group was, in most results, closer to protectivity than the non-phenolic group. Compounds **4, 8**, and **11** were cytotoxic, owing to methyl substitution. This biological difference is due to the dual reactivity of the benzylidene double bond in the structure: the phenolic function allows rearrangements of **5**, **9**, **12**, and **13** for dehydrogenation (hydrogen donation) while the methyl blockade results in thiol addition over the benzylidene double bond (thio-Michael reaction), leading to cytotoxicity [26].

Phenolic OH allows molecules to become antioxidant, whereas the methyl substituent blocks this. This was visible against ROS: non-phenolic **4**, **8**, and **11** did not reduce ROS. Compound **13** with a monocarboxylic function on the central piperidone ring, compound **9** with an alkyl OH, and compound **12** with an NH function at the same position were active antioxidants. For the proposed transition products of the neuroprotection-promoter hydrogen-donor cyclic C_5_-curcuminoids, see [26]. Derivatives **9** and **13** showed an increase in catalase and GPx induction, whereas the non-phenolic compounds remained neutral. This cannot be explained by the hydrogen donor ability. The OH group in the central cyclohexanone ring of **9** and the NH moiety in the piperidone ring of **12** inhibited DNA fragmentation, suggesting a high anti-apoptotic potential in this model. Reduction in microglial activation and glutamate production was observed with compounds **9** and **12**. Phenolic cyclic chalcone **5** exhibited anti-apoptotic potential. The differences in the BBB penetrations of **5**, **9**, **12**, and **13** cannot be explained merely by structural polarity. These curcuminoids are polar due to the presence of phenolic OH, secondary NH, and free carboxylic acid moieties. Binding to BSA and transporter macromolecules in human cells may affect BBB penetration. Further investigations are required to answer these questions.

## 5. Limitations

However, the results are promising and suggest the possibility of developing new therapies for neurodegenerative diseases. The three cyclic C_5_-curcuminoids (compounds **9**, **12**, and **13**) and one cyclic chalcone (compound **5**) require further investigation. The synthetic cyclic C_5_-curcuminoids were tested at individually selected concentrations, highlighting that the observed differences in biological effects are not due to intrinsic compound potency. Instead, these differences may reflect the varying doses used. It is crucial to interpret the results as the effects of each compound at its specific concentration, rather than as a ranking of potency or efficacy. To accurately compare the relative potency of these compounds, future research should employ matched equimolar or equitoxic concentrations and include a compound-vs-compound statistical analysis, which was not part of the current study design. Our studies were limited to in vitro co-culture, utilizing human SH-SY5Y cells and mouse-derived BV-2 microglia. This setup ensured that cytokines secreted by the cells could be specifically quantified, thereby allowing the effects of synthetic cyclic C_5_-curcuminoids on neurons and microglia to be distinguished. It should be noted that the pro-inflammatory cytokine IL-1β was not quantified, precluding the assessment of its impact. Additionally, our experiments were conducted at 24 h intervals, highlighting the necessity for supplementary long-term studies. It is important to further enhance the stability of these compounds through appropriate modifications and formulations, enabling in vivo studies. Investigating and characterizing the oral bioavailability, absorption, and metabolism of curcuminoid derivatives is imperative, as is monitoring the utilization of compounds entering the circulation, their penetration of the blood–brain barrier, and the consequent alterations in biological effects in in vivo models. Furthermore, employing combinations of the compounds under investigation may optimize antioxidant, anti-apoptotic, and anti-inflammatory effects.

## 6. Conclusions

Based on these results, it can be concluded that compounds **5**, **9**, **12**, and **13**, each evaluated at its own individually selected sub-IC50 concentration, are promising candidates for the treatment of neuronal damage in the presence of microglia, which are major contributors to disease progression. At the tested concentration, compound **13** increased catalase and GPx activity and reduced markers of iron-mediated oxidative stress and ferroptosis. Compound **9** also reduced ferroptosis markers and inhibited microglial activation and glutamate synthesis. Compound **5** exhibited consistent anti-apoptotic effects on PARP, cytochrome c, and caspase-9 and 3 markers. Compound **13** reduced the secretion of pro-inflammatory cytokines (IL-6 and TNFα) in both cell types. Because the compounds were tested at different absolute and IC50-relative concentrations, these results describe the effects of each compound under its own tested condition and should not be interpreted as a potency ranking across all compounds. Depending on the prioritized biological property, all four compounds are candidates for further neuroprotective development, ideally at matched concentrations in follow-up studies. The BBB experiments demonstrated the potential for brain penetration in the case of one cyclic C_5_-curcuminoid, compound **12**. The permeability of compounds **5**, **9**, and **13** can be improved through further modifications and formulations, which could facilitate BBB crossing. From a translational perspective, compound **12** is the primary candidate for in vivo follow-up, where brain and plasma exposure and the persistence of the antioxidant and anti-inflammatory effects observed here could be evaluated in a rodent model of the oxidative dopaminergic injury reproduced in vitro by 6-OHDA. In contrast, compounds **5**, **9**, and **13** have limited permeability points, indicating that formulation-based approaches are more suitable than in vivo approaches as the immediate direction. For compound **12**, which showed adequate permeability, the poor aqueous solubility and extensive presystemic metabolism characteristics of curcuminoids would be the primary obstacles to in vivo evaluation, which could be addressed by solid-state and particle-engineering approaches, such as amorphous solid dispersions, nanocrystalline suspensions, cocrystals, or lipid-based delivery systems. In contrast, for compounds **5**, **9** and **13**, the limitation is passive diffusion rather than dissolution and is therefore unlikely to be overcome by solubility enhancement alone.

## Figures and Tables

**Table 1:** Real-time PCR primer list.

Primer	Gene Accession Number	Sequence 5′ → 3′
Iba1 forward	NM_007393.5	GGAAAGTCAGCCAGTCCTCC
Iba1 reverse		CATCACTTCCACATCAGCTTTTGA
β-actin forward	NM_019467.3	CTGTCGAGTCGCGTCCA
β-actin reverse		TCATCCATGGCGAACTGGTG

**Table 2:** Recovery/mass balance of compounds **5**, **9**, **12**, and **13** after the BBB permeability experiments.

Curcuminoid Derivatives	Recovery (%) BBB Model	Amount in Brain Endothelial Cells (%)	Recovery (%) Cell-Free Insert
compound **5**	39.3 ± 2	2.7 ± 1.1	102.8 ± 3.3
compound **9**	37.1 ± 4.4	n.d.	63.4 ± 6.9
compound **12**	51.6 ± 1.2	1.1 ± 0.1	89.5 ± 1.5
compound **13**	70.9 ± 1.6	n.d.	66.7 ± 3.1

n.d., not detected (below LOD/LOQ).

## Data Availability

The original contributions presented in this study are included in the article/Supplementary Materials. Further inquiries can be directed to the corresponding author.

## Supplementary Materials

The following supporting information can be downloaded at https://www.mdpi.com/article/10.3390/antiox15091085/s1: Supplementary File S1: Quantification of curcuminoid derivatives. Supplementary Figure S1: Efficiency, melting curves, and peaks of Iba1 and β-actin obtained from CFX Maestro 2.3 Software. Supplementary File S2: Densitometry data of WB.

## Abbreviations

The following abbreviations are used in this manuscript:

6-OHDA	6-hydroxydopamine
AGC	automatic gain control
ATRA	all-trans retinoic acid
BBB	blood–brain barrier
BSA	bovine serum albumin
Cas-3	caspase-3
CX3CR1	fractalkine receptor
Cyt c	cytochrome c
DMSO	dimethyl sulfoxide
EBA	Evans blue-labeled albumin
EIC	extracted ion chromatogram
ELISA	Enzyme-Linked Immunosorbent Assay
FBS	fetal bovine serum
FKN	fractalkine
GAPDH	glyceraldehyde 3-phosphate dehydrogenase
GPx	glutathione peroxidase
GR	glutathione reductase
GSH	glutathione
GSSG	oxidized glutathione
H_2_O_2_	hydrogen peroxide
hEC	human endothelial cells
HESI	heated electrospray ionization
HPLC	high-performance liquid chromatography
IAP	inhibitors of apoptosis
Iba1	Ionized calcium-binding adapter molecule 1
IL	interleukin
JAK	Janus kinase
LOD	limit of detection
LOQ	limit of quantification
MAO-B	monoamine oxidase B
MAPK	mitogen-activated protein kinase
MDA	malondialdehyde
MDR	multiple-drug resistance
MS	mass spectrometry
NFκB	nuclear factor kappa-light-chain-enhancer of activated B cells
NMR	nuclear magnetic resonance
Papp	apparent permeability coefficients
PARP	poly ADP-ribose polymerase
P/S	penicillin/streptomycin
PBS	phosphate-buffered saline
PD	Parkinson’s disease
PCs	pericytes
ROS	reactive oxygen species
RTCA-SP	Real-Time Cell Analysis—single plate
SAR	structure–activity relationship
SF	sodium fluorescein
SOD	superoxide dismutase
STAT	signal transducer and activator of transcription
TAC	total antioxidant capacity
TNFα	tumor necrosis factor α
