# Supplementary material for: Synthetic Cyclic C5-Curcuminoids Mitigate Oxidative, Apoptotic and Inflammatory Damage in a 6-OHDA-Induced Neuron–Microglia Co-Culture Model of Neuronal Damage
Source: Antioxidants (Basel). 2026 Aug 29;15(9):1085. doi: 10.3390/antiox15091085 (PMC13603456; doi:10.3390/antiox15091085)

Original Western blots

Figure 16A: differentiated SH-SY5Y cells; in the order of appearance in the figure

p105/p50; (105 and 50 kDa) 10% SDS-PAGE

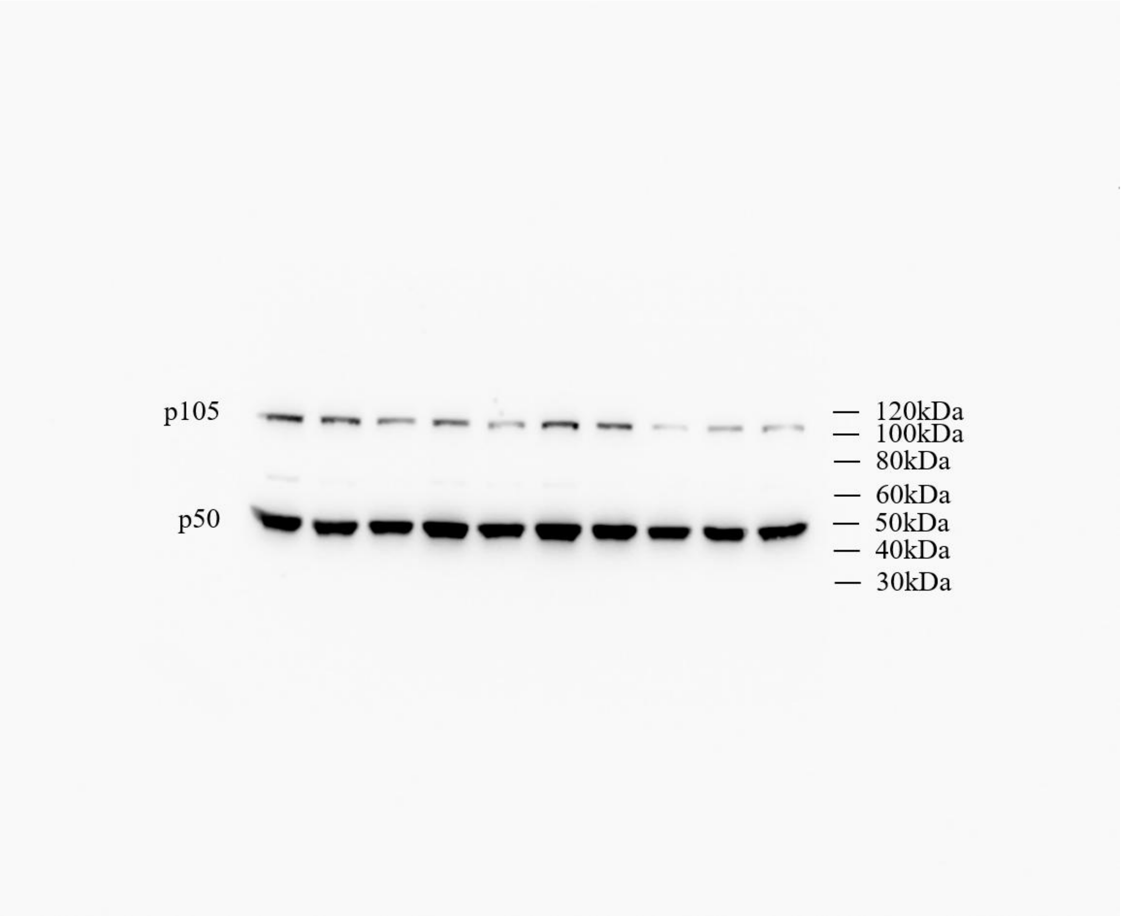

p105/**p50** (105 and 50 kDa) 10% SDS-PAGE

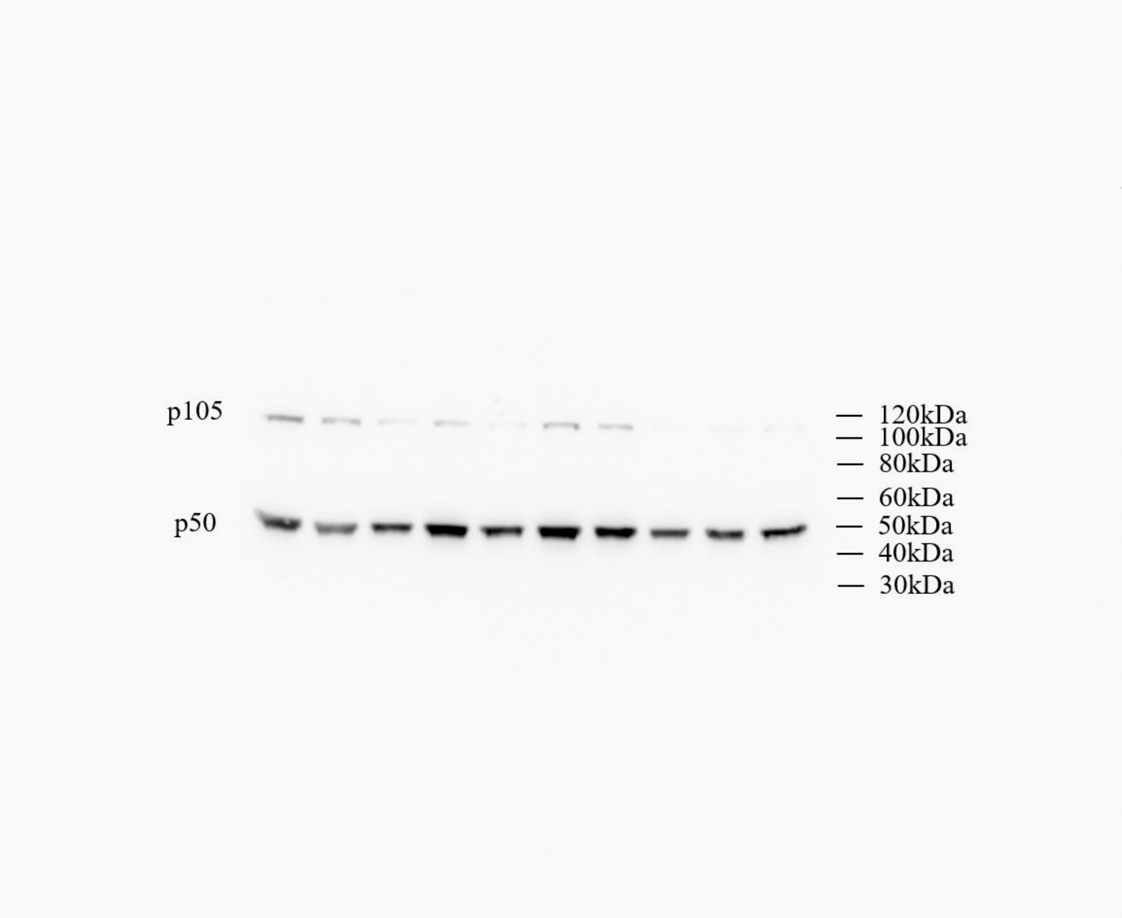

P-p65 (65 kDa) 10% SDS-PAGE

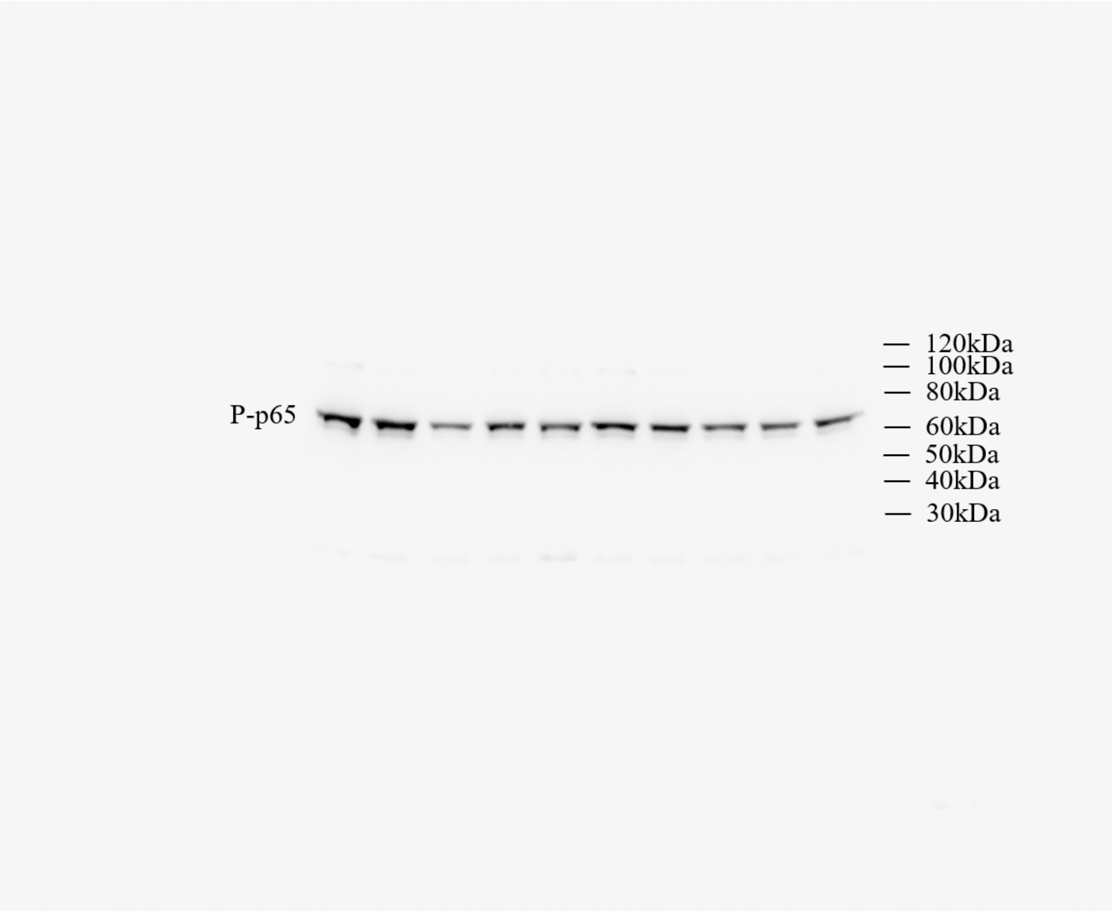

GAPDH (38 kDa)10% SDS-PAGE

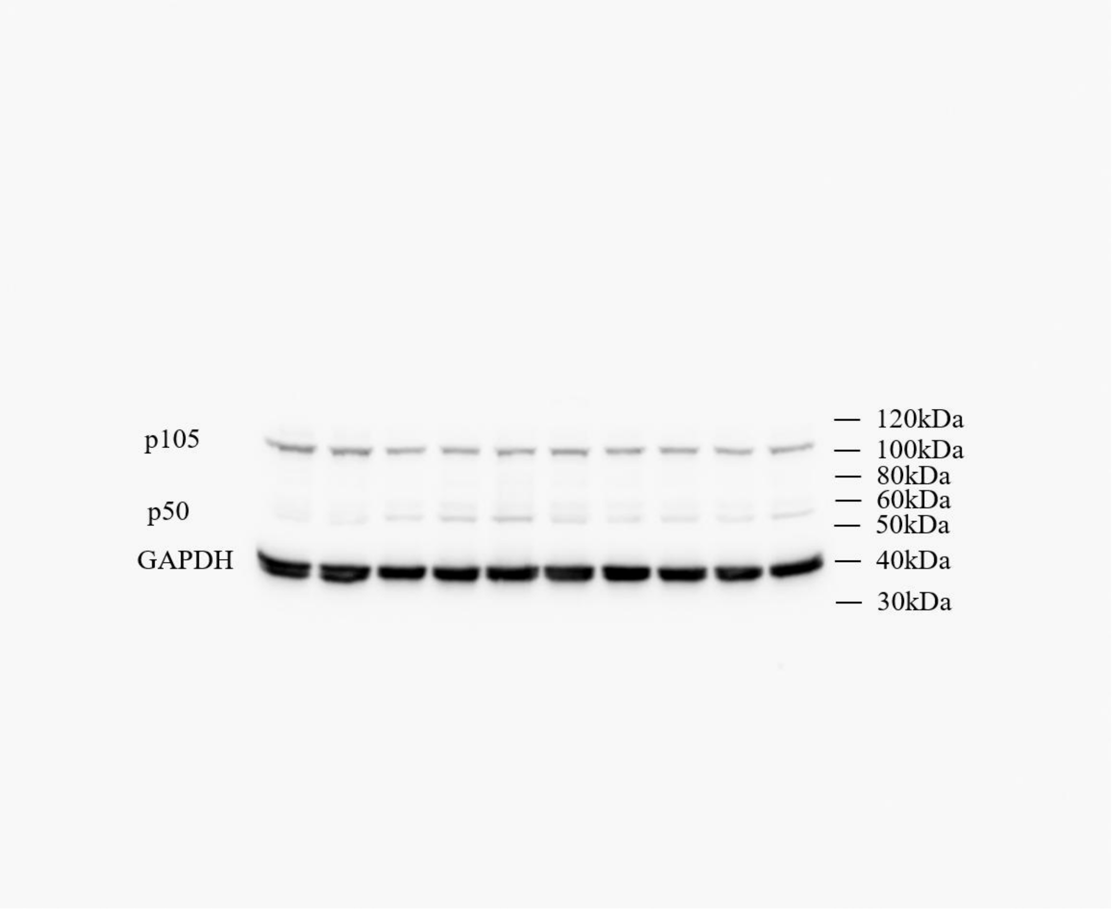

Figure 16D: BV-2 microglia cells; in the order of appearance in the figure

p105 (105 kDa) 10% SDS-PAGE

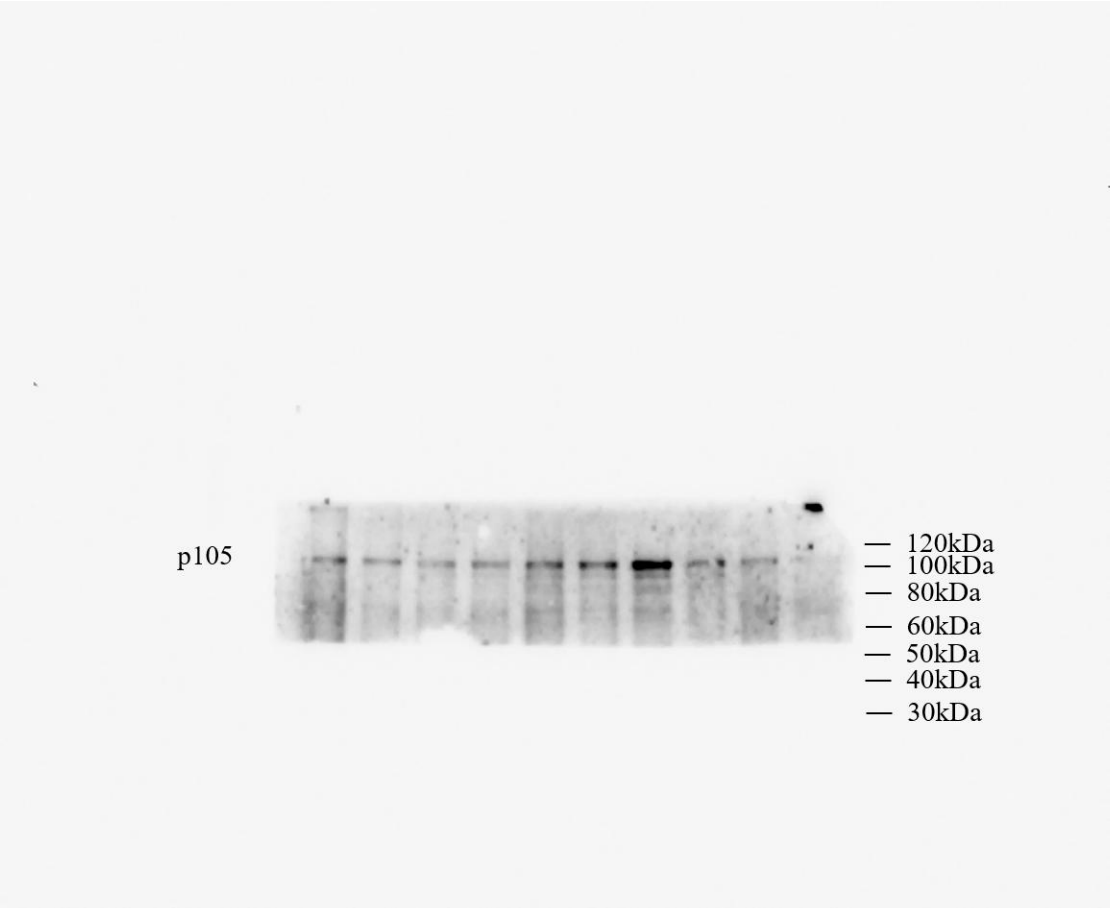

p50 (50 kDa) 10% SDS-PAGE

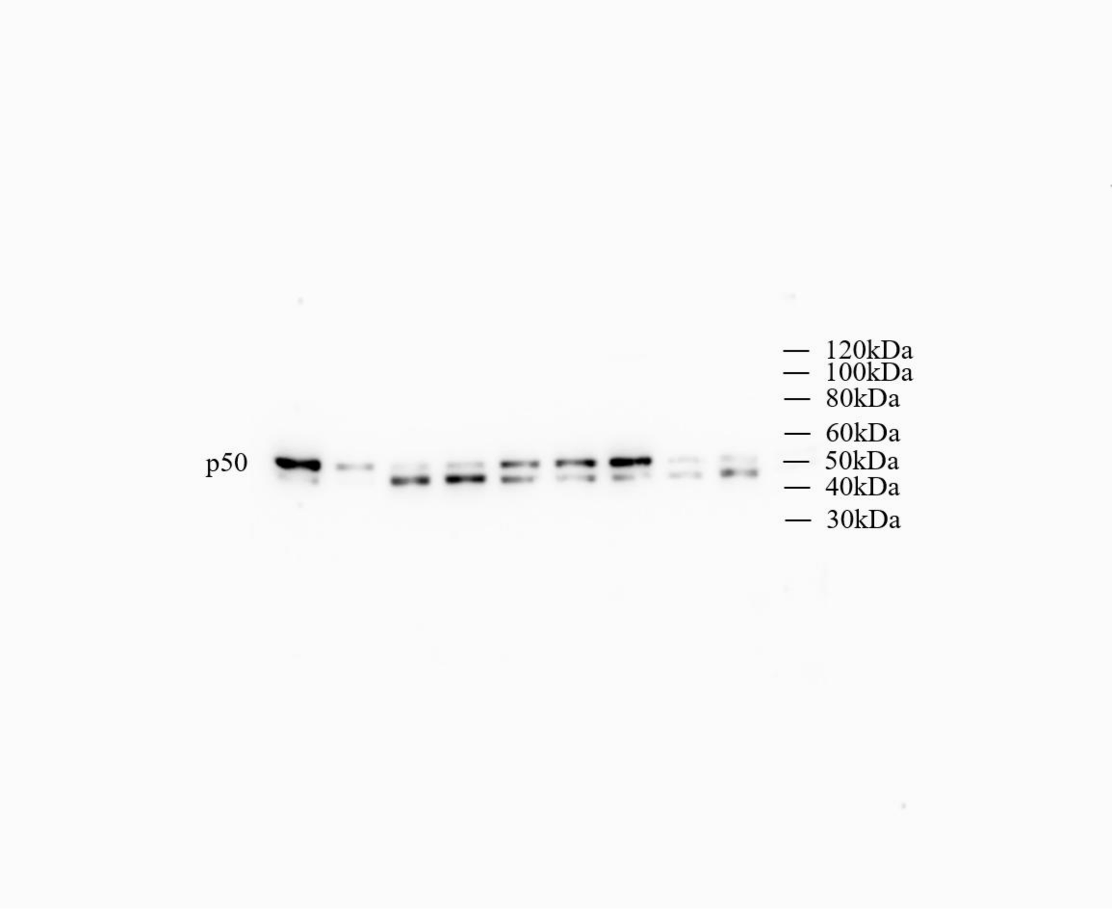

P-p65 (65 kDa) 10% SDS-PAGE

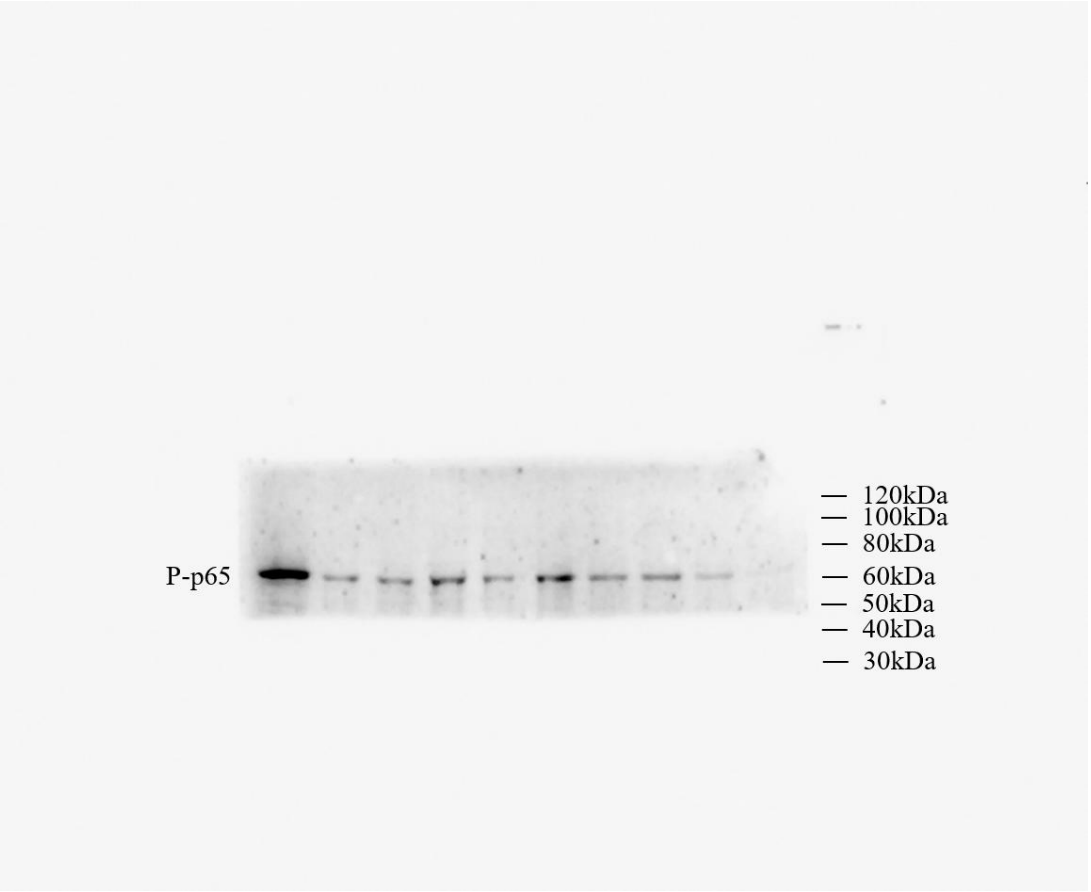

β-actin (42 kDa) 10% SDS-PAGE

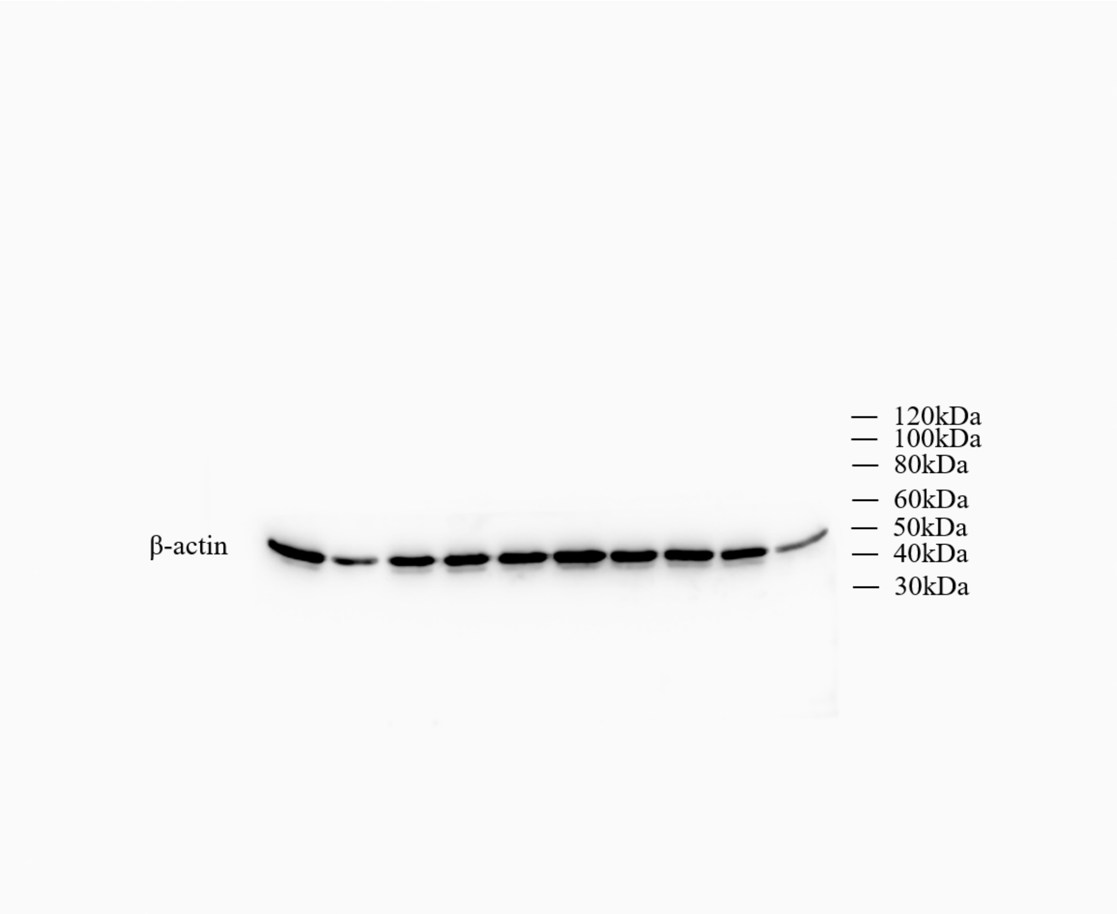

Supplement: Supplementary file 1 [file antioxidants-15-01085-s001.zip › Original Western blots.pdf]
