# Supplementary material for: Synthetic Cyclic C5-Curcuminoids Mitigate Oxidative, Apoptotic and Inflammatory Damage in a 6-OHDA-Induced Neuron–Microglia Co-Culture Model of Neuronal Damage
Source: Antioxidants (Basel). 2026 Aug 29;15(9):1085. doi: 10.3390/antiox15091085 (PMC13603456; doi:10.3390/antiox15091085)
Supplement: Supplementary file 1 [file antioxidants-15-01085-s001.zip › Supplementary Figure S1 Efficiency, melting curves, and peaks of Iba1 and β-actin obtained from CFX Maestro 2.3 Software.pdf]

A

Target Names

Name	Full Name	Reference	Auto Efficiency	Efficiency
Actin	Actin	True	Yes	100.00%
Iba1	Iba1	False	Yes	100.00%

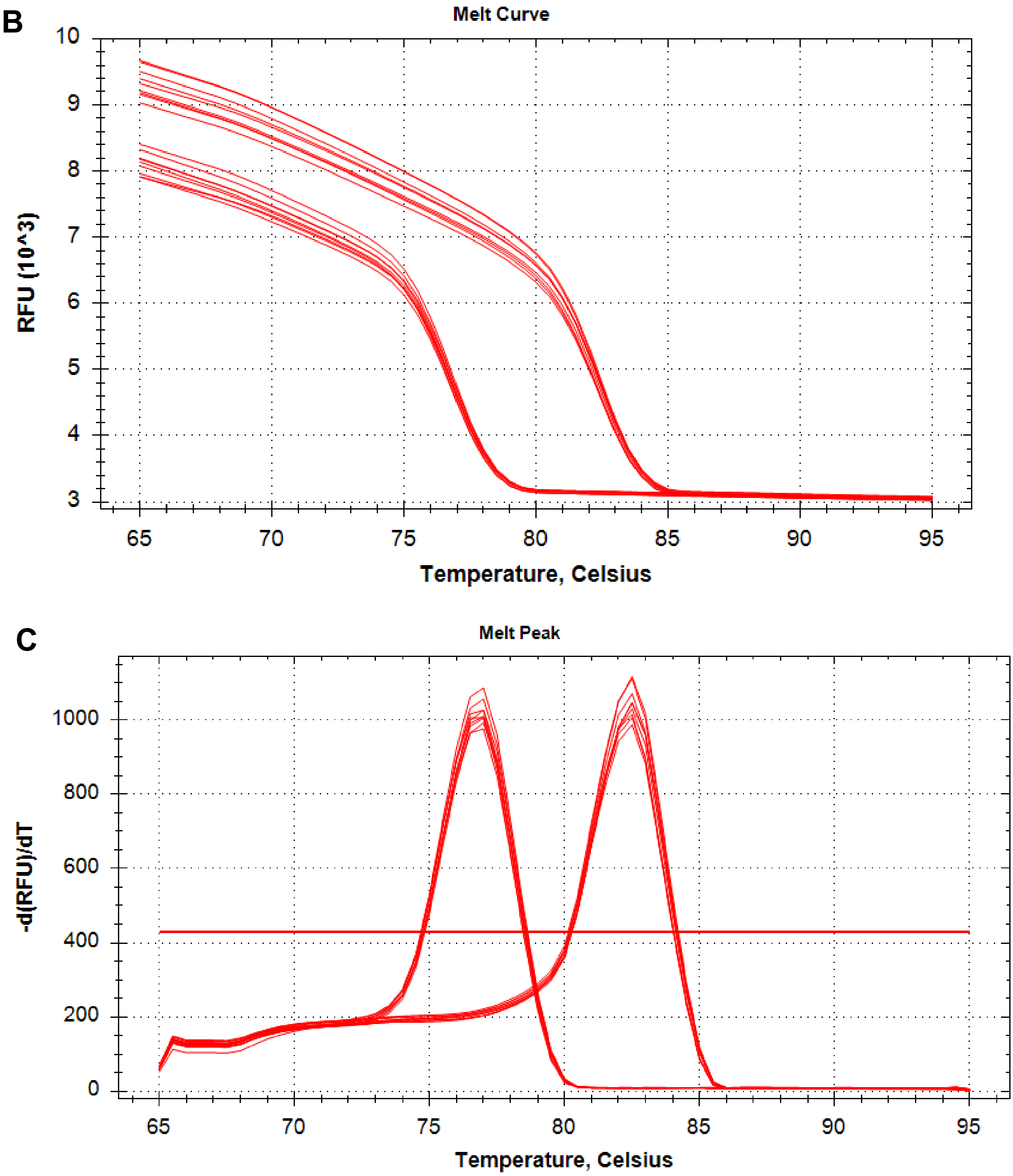

Supplementary Figure S1. Efficiency (A), melting curves (B), and peaks (C) of Iba1 and β-actin obtained from CFX Maestro 2.3 Software. Melting TM for β-actin: 82.5 ^{0}C; Melting TM for Iba1: 77 ^{0}C
