# Supplementary material for: Synthetic Cyclic C5-Curcuminoids Mitigate Oxidative, Apoptotic and Inflammatory Damage in a 6-OHDA-Induced Neuron–Microglia Co-Culture Model of Neuronal Damage
Source: Antioxidants (Basel). 2026 Aug 29;15(9):1085. doi: 10.3390/antiox15091085 (PMC13603456; doi:10.3390/antiox15091085)
Supplement: Supplementary file 1 [file antioxidants-15-01085-s001.zip › Supplementary File S1 Quantification of curcuminoid derivatives.pdf]

Synthetic cyclic C5-curcuminoids mitigate oxidative, apoptotic, and inflammatory damage in a 6-OHDA-induced neuron-microglia co-culture model of neuronal damage

Edina Pandur [1]*, Zsombor Bence Tóth [1], Levente Tyukodi [2], Ilona Gróf [3], Szilvia Veszelka [3], Mária A. Deli [3], Zsuzsanna Rozmer [2] and Imre Huber [2]

[1] Department of Pharmaceutical Biology, Faculty of Pharmacy, University of Pécs, Rókus str. 4, H-7624, Pécs, Hungary; pandur.edina@gytk.pte.hu; bencetoth1025@gmail.com

[2] Institute of Pharmaceutical Chemistry, Faculty of Pharmacy, University of Pécs, Rókus str. 4, H-7624, Pécs, Hungary; tyukodi.levente@pte.hu; rozmer.zsuzsanna@pte.hu; imre.huber@aok.pte.hu

[3] 3Institute of Biophysics, HUN-REN Biological Research Centre, Temesvári Krt. 62, H-6726, Szeged, Hungary; grof.ilona@brc.hu; veszelka.szilvia@brc.hu; deli.maria@brc.hu

* Correspondence: pandur.edina@gytk.pte.hu

Quantification of the curcuminoid derivatives

The concentrations of compound **5**, compound **9**, compound **12,** and compound **13** in the donor, acceptor, cell-lysate, and cell-free insert samples were determined by external calibration from the extracted ion chromatogram of each protonated molecule (see LC-MS method). For every derivative, a seven-point calibration series (25, 50, 100, 200, 400, 700, and 1000 nM) was measured on three independent occasions, and two regression models were applied. Sample concentrations were back-calculated from a full-range (25-1000 nM) weighted least-squares calibration line ($1/x^2$ weighting, with each replicate treated as an individual data point) as $x = (y - a)/b$, where a and b are the intercept and slope of the line. The $1/x^2$ weighting was used to accommodate the heteroscedastic response typical of LC-MS detection and provided a better fit across the full range than an unweighted model (full-range $R^2 = 0.96$-0.99).

The limit of detection (LOD) and the limit of quantification (LOQ) were derived from a separate calibration line restricted to the lower concentration range (25-200 nM), which better reflects the response variability near the quantification limit, as $LOD = 3.3 \times s_{y/x} / b$ and $LOQ = 10 \times s_{y/x} / b$, where $s_{y/x}$ is the residual standard deviation of the lower-range line and b its slope [1]. A single full-range model was not used for LOD/LOQ estimation because the $1/x^2$ weighting shifts the fitted slope and intercept towards higher-concentration points, thereby underestimating the residual variance near the quantification limit. Restricting the calibration line to the lower range (25–200 nM) provides a more conservative and analytically appropriate estimate of the response variability in the region where LOD and LOQ are defined. The lower-range lines were highly linear ($R^2 \geq 0.998$), giving LOD values of 6.7-13.5 nM and LOQ values of 20.3-40.8 nM (Supplementary Table S1).

Supplementary Table S1. Calibration and method performance parameters for the four curcuminoid derivatives.

**Curcuminoid derivative**	**LOD (nM)**	**LOQ (nM)**	**Lower-range line, 25-200 nM (R^2)**	**Full-range WLS line, $1/x^2$, 25-1000 nM (R^2)**
compound 5	9.4	28.5	$y = 98\,330 \cdot x - 828\,101$ (0.9991)	$y = 104\,955 \cdot x - 900\,988$ (0.9637)
compound 9	13.5	40.8	$y = 157\,294 \cdot x - 312\,478$ (0.9982)	$y = 155\,664 \cdot x - 176\,643$ (0.9882)

compound 12	7.3	22.0	y = 182 520·x - 541 690 (0.9995)	y = 193 554·x - 1 117 185 (0.9768)
compound 13	6.7	20.3	y = 85 048·x + 143 608 (0.9995)	y = 86 751·x + 128 098 (0.9678)

System suitability was monitored throughout each sequence from the mass accuracy of every $[M+H]^+$ (within ±5 ppm of the theoretical value) and the stability of the retention times. An exploratory precision and accuracy check of the back-calculated calibration replicates (CV and bias per level) was also carried out; given the limited replication (n = 3 per level), these results are considered indicative rather than constituting a full bioanalytical validation.

A

Target Names

Name	Full Name	Reference	Auto Efficiency	Efficiency
Actin	Actin	True	Yes	100.00%
Iba1	Iba1	False	Yes	100.00%

B

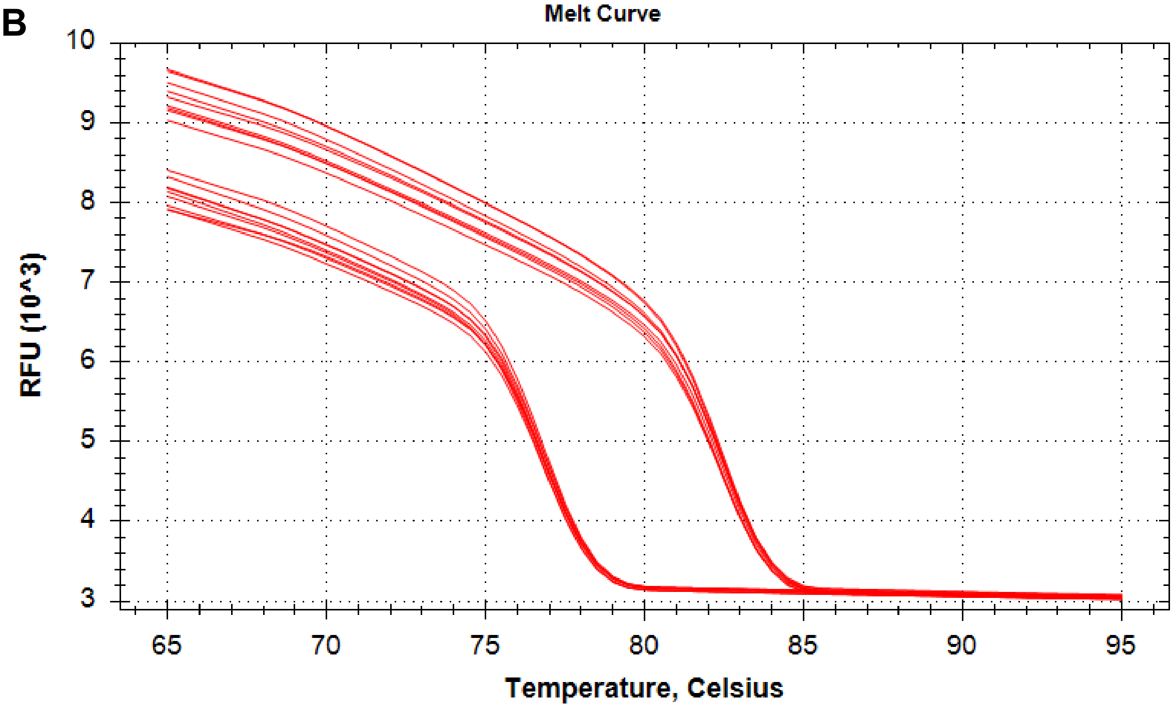

C

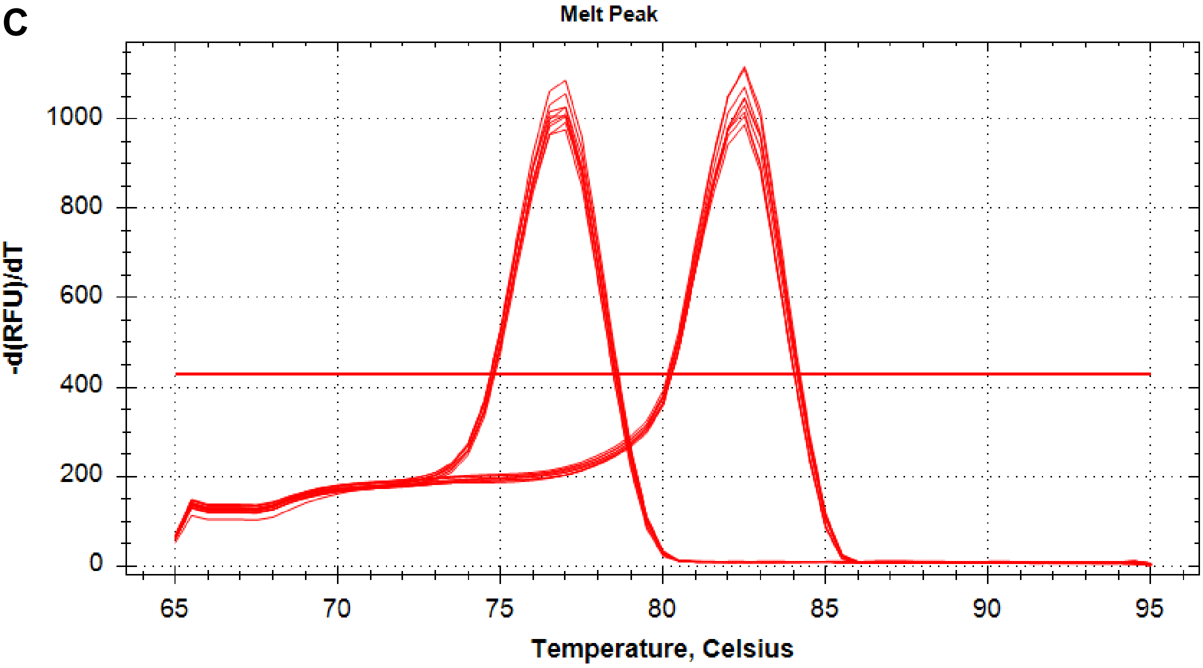

Supplementary Figure S1. Efficiency (A), melting curves (B), and peaks (C) of Iba1 and β-actin obtained from CFX Maestro 2.3 Software. Melting TM for β-actin: 82.5 ⁰C; Melting TM for Iba1: 77 ⁰C
